# Fate of the human Y chromosome linked genes and loci in prostate cancer cell lines DU145 and LNCaP

**DOI:** 10.1186/1471-2164-14-323

**Published:** 2013-05-11

**Authors:** Sandeep Kumar Yadav, Anju Kumari, Sher Ali

**Affiliations:** 1 Molecular Genetics Laboratory, National Institute of Immunology, Aruna Asaf Ali Marg, New Delhi 110067, India

**Keywords:** Prostate cancer, DU145, LNCaP, MSY, STS, DYZ1

## Abstract

**Background:**

Prostate cancer is a known cause of mortality in men worldwide although the risk factor varies among different ethnic groups. Loss of the Y chromosome is a common chromosomal abnormality observed in the human prostate cancer.

**Results:**

We screened 51 standard sequence tagged sites (STSs) corresponding to a male-specific region of the Y chromosome (MSY), sequenced the coding region of the *SRY* gene and assessed the status of the DYZ1 arrays in the human prostate cancer cell lines DU145 and LNCaP. The MSY was found to be intact and coding region of *SRY* showed no sequence variation in both the cell lines. However, DYZ1 arrays showed sequence and copy number variations. DU145 and LNCaP cells were found to carry 742 and 1945 copies of the DYZ1, respectively per 3.3 pg of genomic DNA. The DYZ1 copies detected in these cell lines are much below the average of that reported in normal human males. Similarly, the number of “TTCCA” repeat and its derivatives within the DYZ1 arrays showed variation compared to those of the normal males.

**Conclusions:**

Clearly, the DYZ1 is maximally affected in both the cell lines. Work on additional cell lines and biopsied samples would augment our understanding about the susceptibility of this region. Based on the present work, we construe that copy number status of the DYZ1 may be exploited as a supplementary prognostic tool to monitor the occurrence of prostate cancer using biopsied samples.

## Background

The incidence of prostate cancer (PC) is increasing worldwide though varies amongst different ethnic groups [1]. It is a common malignancy affecting global population of men and is a significant cause of morbidity and mortality. The prevalence of this cancer is highest in the Western countries and lowest in the Asian countries [2]. Despite its high prevalence, very little is known about the molecular mechanism of its tumorigenesis.

Reports are there suggesting the involvement of chromosomes 1, 5, 6, 7, 8, 10,12, 13, 17,18, 20, X and the Y encompassing several genetically susceptible loci [3] though their precise roles still remain unclear. The advanced stage of PC shows complex chromosomal changes accumulated during the tumour progression [3-7]. Loss of the Y chromosome is a common chromosomal abnormality observed in the human PC [8] and this occurs only in neoplastic, but not in stromal cells [9]. Loss of genetic material may lead to the loss of putative tumour suppressor genes, which will ultimately lead to cancer. This hypothesis is supported by the fact that a normal human Y chromosome transferred to a Y chromosome lacking PC cell line PC3, suppressed its tumorigenic property [8]. Genetic predisposition of certain Y chromosomal haplogroups towards PC strongly suggests a positive correlation [1,10,11]. However, study conducted on Korean population showed lack of association between Y chromosomal haplogroups and PC [12]. The involvement of several Y linked genes and loci have been reported to be associated with the progression of PC [13,14]. The male sex determining gene *SRY* is down regulated in PC and is a negative regulator of the androgen receptor [15]. *SRY* does not express in any adult male tissue except the adult testis. However, *SRY* is expressed in DU145 [16], androgen untreated LNCaP cells [13] and prostate adenocarcinoma cell lines. Thus, *SRY* gene seems to play an important role for the development and progression of PC.

The human Y chromosome is male specific, constitutively haploid, passed on from father to son and largely escapes meiotic recombination. Approximately, 95% (60 Mb) of the human Y chromosome represents non recombining region of Y (NRY), also known as MSY. Similarly, 5% (3 Mb) of the Y-chromosome is composed of pseudo-autosomal region (PAR) necessary for the pairing with the sex chromosomes. There are 1287 MSY specific STS markers encompassing the human Y chromosome with an average spacing of 14 Kb spanning over 1698 loci [17]. Deletion mapping based on the STSs has advanced our understanding of the Y chromosome structure and functions. Previous studies were mostly conducted on three commercially available PC cell lines LNCaP, PC3 and DU145 using chromosome banding techniques [18], Fluorescence *in situ* hybridization (FISH) [19], Comparative genomic hybridization (CGH) [20], and Spectral karyotyping (SKY) [21,22]. However, none of these techniques provided evidence towards the involvement of Y chromosome linked genes and loci. In the present study, we studied DU145 (originated from the metastatic site brain of a 69 years old Caucasian male) and LNCaP (originated from the metastatic site left supraclavicular lymph node of a 50 years old Caucasian male) cell lines using 51 STSs specific to MSY. DU145 is Hypotriploid having both 61 and 62 chromosomes with highest rate of occurrence and carries single Y per cell. The Y chromosome of DU145 carries a translocated part of chromosome 20 [3]. LNCaP is hypotetraploid having 84 chromosomes occurring in 22% of the cells [23] and carries 2 Y chromosomes per cell [3].

Approximately, 20% of the human Y chromosome harbours DYZ1 satellite sequence [24]. DYZ1 was identified as 3.4 Kb band generated on *Hae*III digestion of the human male genomic DNA [25]. A normal human Y chromosome contains approximately 4000–4300 copies of the DYZ1 arrays [26]. Since DYZ1 copies do not participate in recombination, it was deduced to have no functional or evolutionary advantage [27]. However, this is now reported to play a crucial role in chromatin folding and maintenance of the structural integrity of the Y chromosome [26].

With this background, we studied status of several Y linked genes and loci and assessed copy number variation of DYZ1 arrays using real time PCR in DU145 and LNCaP cell lines. In addition, we sequenced the 3564 bp unit of DYZ1 array and the coding region of *SRY* gene from these cell lines.

## Results

### Status of the MSY region in DU145

**Figure 1:**
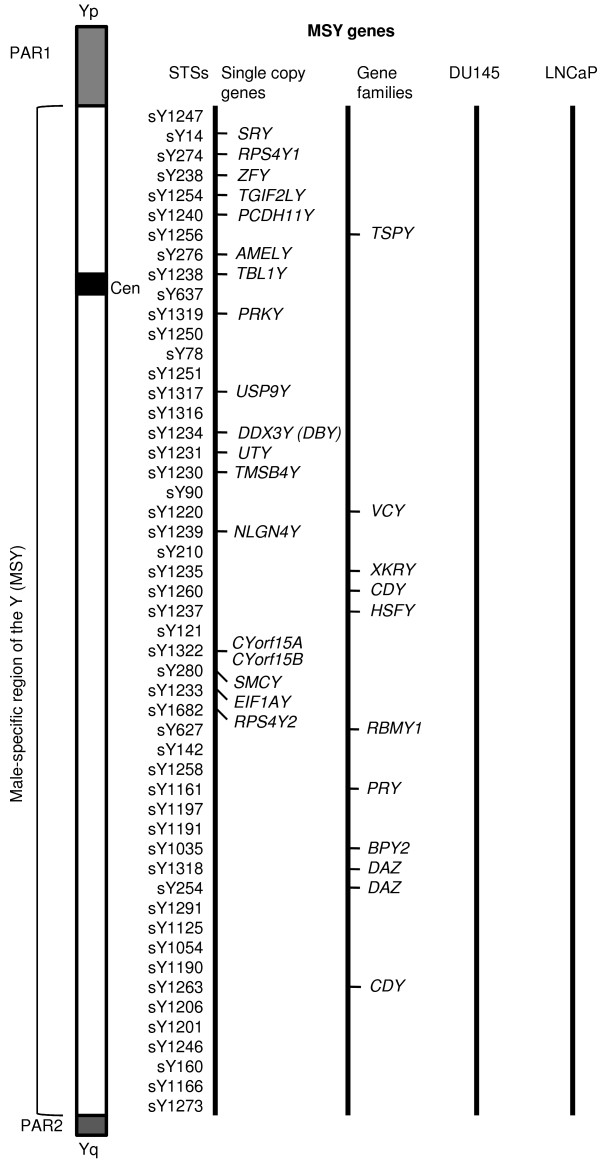
**Schematic representation of the human Y chromosome showing MSY region and positions of 51 STSs screened in DU145 and LNCaP cells.** Single copy genes, gene families and their corresponding STSs are shown on the vertical lines adjacent to the diagram of Y chromosome. The vertical line on the right most side of the picture is showing presence of all the 51 STSs studied.

The genomic DNA from DU145 and LNCaP was screened for the presence or absence of 51 STSs specific to MSY (Figure 1 and Table 1). These STSs in both the cell lines were found to be intact.

**Table 1:** List of STSs analyzed to ascertain the genetic integrity of the Y-chromosome in the prostate cancer cell lines DU145 and LNCaP

**Serial No.**	**STS Identifier**	**Location**	**Multi-Copy**	**STS Status in DU145**	**STS status in LNCaP**
1	sY14	*SRY* exon 1		+	+
2	sY1240	*PCDH11Y* intron 3		+	+
3	sY276	*AMELY* exon 4/intron 4		+	+
4	sY1238	*TBL1Y* exon 11		+	+
5	sY637	*PRKY* 5’ upstream of gene		+	+
6	sY1319	*PRKY* 3’ UTR		+	+
7	sY1250	Proximal boundary of *TSPY* array		+	+
8	sY78	Alpha satellite (*DYZ3*) sequences in centromeric region	*	+	+
9	sY1251	Boundary between centromere and Yq		+	+
10	sY1317	*USP9Y* exon 3		+	+
11	sY1316	*USP9Y* exon 26		+	+
12	sY1234	*DDX3Y* (*DBY*) exon 9		+	+
13	sY1231	*UTY* exon 8		+	+
14	sY1235	*XKRY* exon 1	*	+	+
15	sY1260	*CDY2* exon 1	*	+	+
16	sY1237	*HSFY* exon 2	*	+	+
17	sY121	Immediately distal to palindrome P4		+	+
18	sY1322	Between *CYorf15A & CYorf15B*		+	+
19	sY280	*JARID1D* (*SMCY*) exon 9/intron 9		+	+
20	sY1233	*EIF1AY* exon 1		+	+
21	sY1682	*RPS4Y2* exon 1		+	+
22	sY627	*RBMY1* exon 12	*	+	+
23	sY1258	Boundary between unique sequence u1 & blue amplicon b1 in *AZFc*		+	+
24	sY1161	*PRY* intron 2	*	+	+
25	sY1197	Internal boundary of Palindrome P3		+	+
26	sY1191	Unique sequence u3 in *AZFc*		+	+
27	sY1035	*BPY2* intron 5	*	+	+
28	sY1318	*DAZ* exon 11	*	+	+
29	sY254	*DAZ* exon 3	*	+	+
30	sY1291	Red/gray boundary in *AZFc*		+	+
31	sY1125	Blue/gray boundary in *AZFc*	*	+	+
32	sY1054	Blue/yellow boundary in *AZFc*	*	+	+
33	sY1190	Yellow amplicon in *AZFc*	*	+	+
34	sY1263	*CDY1* exon 1/intron 1	*	+	+
35	sY1206	Yellow/green boundaries in *AZFc*	*	+	+
36	sY1201	Distal boundary of gray amplicon in *AZFc*		+	+
37	sY1256	*TSPY* intron 5	*	+	+
38	sY1247	Boundary between PAR1 and *MSY*		+	+
39	sY1230	*TMSB4Y* exon 1/intron 1		+	+
40	sY1166	In MSY between distal Yq heterochromatin & PAR2		+	+
41	sY274	*RPS4Y1* intron 4		+	+
42	sY1220	*VCY* exon 2	*	+	+
43	sY90	*KALP* intron 1		+	+
44	sY210	*STSP* intron 5		+	+
45	sY1254	*TGIF2LY* exon 1		+	+
46	sY1239	*NLGN4Y* exon 1		+	+
47	sY1273	Near boundary between MSY & PAR2		+	+
48	sY238	*ZFY* intron 2		+	+
49	sY1246	Proximal portion of distal Yq heterochromatin		+	+
50	sY142	Proximal to *AZFc*		+	+
51	sY160	Satellite-3 (*DYZ1*) sequences in distal Yq heterochromatin	*	+	+

Multi-copy STSs are indicated by star (*) and plus (+) sign denotes the presence of a particular STS.

### HERV15 provirus sequence recombination

**Figure 2:**
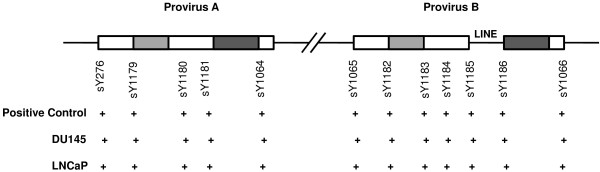
**Deletion breakpoints mapping of *AZFa *region in DU145 and LNCaP based on STS analysis.** Schematic diagram showing positions of proviruses **A** and **B**. Light and dark grey blocks are the regions of identical sequences in the proviruses **A** and **B** involved in recombination. 12 STSs specific to proviruses A and B are shown along with the corresponding positive controls.

Human *AZFa* region contains HERV15 provirus A and B sequences and homologous recombination between these two regions accounts for most of the *AZFa* deletions [28]. The *AZFa* regions of DU145 and LNCaP Y chromosomes were found to remain intact showing the presence of *USP9Y* and *DBY* genes (Figure 2).

### Interstitial deletion mapping

**Figure 3:**
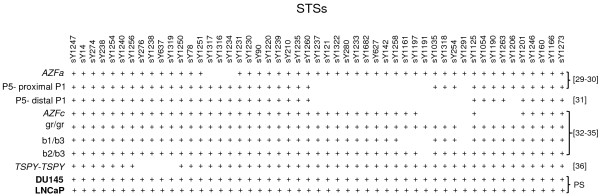
**STS map showing common interstitial deletions of the human Y chromosome based on different studies conducted earlier.** Plus sign and blank spaces denote the presence and absence of a particular STS, respectively. The corresponding references related to these studies are given on the right [see references [29-36]. Last row shows the results of the present study denoted as PS.

There are known deletion patterns observed in different clinical conditions e.g. *AZFa,* P5- proximal P1, P5- distal P1, *AZFc*, gr/gr, b1/b2 and b2/b3 and *TSPY*-*TSPY* deletion [29-36]. We have screened STSs encompassing these regions which were found to remain intact in DU145 of LNCaP cells (Figure 3).

### *DAZ* SNV analysis

The normal Y chromosome harbours four copies of *DAZ* gene [37]. The SNV analysis of *DAZ* was conducted using appropriate enzymes [38] to digest PCR products (Table 2). The *DAZ* region in DU145 and LNCaP showed presence of both alleles A and B for the SNVs (*DAZ*-SNV_I, *DAZ*-SNV_II, *DAZ*-SNV_III, *DAZ*-SNV_IV, *DAZ*-SNV_V, *DAZ*-SNV_VII, *GOLY*-SNV_1, *TTTY4*) screened. For *DAZ*-SNV_VI and *BPY2,* DU145 showed both the alleles A and B while LNCaP had only allele A (Table 2).

**Table 2:** **Details of the SNVs studied for *DAZ*, *GOLY*, *BPY2 *and *TTTY4 *genes**

**Target**	**SNV**	**Oligos used**	**Accession no.**	**Product size**	**Enzyme for digestion**	**Restriction site**	**Fragments**	**Alleles**	**Present in copies**	**DU145**	**LNCaP**
	*DAZ*-SNV_I	SA 770 CACAGGCACTCAGTAACTATCTC	*G73167*	709	*Fsp1*	TGC/GCA	709	A	1,2,3 4	A&B	A&B
							398 + 311	B			
		SA771CAGTGTTTCACCCACCACTTCTGGGT									
	*DAZ*-SNV_II	SA 446 GACATCCACGTCATTAACAAACG	*G73166*	182	*Mbo1*	/GATC	182	A	1 2,3,4	A&B	A&B
							122 + 60	B			
		SA 447 GGAAGCTGCTTTGGTAGATAC									
	DAZ-SNV_III(sY586)	SA 772 GTGTGGCACATATGCCTATAAA	*G63907*	301	*Taq1*	T/CGA	301	A	2 1,3,4	A&B	A&B
							184 + 117	B			
		SA 773 TTGGTACATCCAGATGCAGAT									
***DAZ *gene**	*DAZ*-SNV_IV	SA 774 CTTCCTCATCTTTCTTGACTT	*G73168*	630	*AluI*	AG/CT	630	A	2 1,3,4	A&B	A&B
							398 + 262	B			
		SA 775 TTATTTATTCCTCAAAAAGGTG									
	DAZ-SNV_V (sY587)	SA 444 TGGTTAATAAAGGGAAGGTGTTTT	*G63908*	244	*DraI*	TTT/AAA	195 + 49	A	3,4 1,2	A&B	A&B
							122 + 73 + 49	B			
		SA 445 TCTCCAGGACAGGAAAATCC									
	*DAZ*-SNV_VI	SA 776 GGGCCTAGTCTCTAGATCATT	*G73169*	431	*AflIII*	A/CRYGT	431	A	1,2,3 4	A&B	A
							248 + 183	B			
		SA 777 GCTAGAACCAAATATTCTGGAT									
	DAZ-SNV_VII (sY581)	SA 442 CACTGCCCTAATCCTAGCACA	*G63906*	252	*Sau3AI*	/GATC	189 + 63	A	1,4 2,3	A&B	A&B
							130 + 63 + 59	B			
		SA 443 TCTTCTGGACATCCACGTCA									
***GOLY1***	*GOLY*-SNV_1	SA768 TTGGCCTGTTGCTTCTAGGGTT	*BV012733*	531	*HhaI*	GCG/C	531	A	1 Copy	A&B	A&B
		SA769 ACAGGGAGGGTGCTGTCACA					289 + 282	B	1 Copy		
***BPY2***	*BPY2*	SA766 AAGCCCATTGCTGAGATACTG	*BV012732*	470	*EcoRV*	GAT/ATC	470	A	2 Copy	A&B	A
		SA767 TTGTGATTCTGACCCAACGA					289 + 181	B	1 Copy		
***TTTY4***	*TTTY4*	SA764 TGCAGACAGCACTGTGGCTT	*BV012731*	541	*HaeIII*	GG/CC	541	A	1 Copy	A&B	A&B
							323 + 218	B	2 Copy		
		SA765 GTATATGGCATAATTTCACCTG									

### *SRY* gene sequence

We amplified 824 bp fragment of the *SRY* exon having 615 bp long coding fragment (Table 3B), cloned and sequenced. The sequences of the *SRY* gene in both DU145 and LNCaP were found to be intact (see Additional file 1).

**Table 3:** List of primers used for PCR amplification of DYZ1 array (A) and SRY gene (B)

**(A)**					
**Serial No.**	**Primer ID**	**Primer Sequence**	**Length (bp)**	**Location (DYZ1 array)**	**Orientation**
**1**	SAS 1	CCATTCGAGACCGTAGCAAT	20	35-16 (5’ upstream to HaeIII site)	5’-3’
**2**	SAS 2	TTTCCTTTCGCTTGCATTCCAT	22	63-84	5’-3’
**3**	SAS 3	ATTTGATGCCATCCCATGAC	20	763-782	5’-3’
**4**	SAS 4	TTTTGAGTCCGTTCCATAACAC	22	1380-1401	5’-3’
**5**	SAS 5	TCCTTTGCCTTCCATTCG	18	1668-1685	5’-3’
**6**	SAS 6	TGCAGTCTTTTCCCTTCGAG	20	2564-2583	5’-3’
**7**	SAS 7	ATTGGATGGGATTGGAATGA	20	861-880	3’-5’
**8**	SAS 8	TGGATGGACTGCAATAGAAAG	22	1600-1621	3’-5’
**9**	SAS 9	TCGAATGGAAGGCAAAGG	18	1669-1686	3’-5’
**10**	SAS 10	CGACTGGTACGGACTCCAAT	20	2637-2656	3’-5’
**11**	SAS 11	GACTGGAAAGGCTGGGTGTCGA	22	3419-3440	3’-5’
**12**	SAS 12	TGGACAGCCTGGAATAAAGTG	21	3586-3606	3’-5’
**(B)**					
**1**	SA 531	GAATCTGGTAGAAGTGAGTTTTGGA	25	61-85	5’-3’
**2**	SA 532	GCCTTTATTAGCCAGAGAAAAGAAA	25	860-884	3’-5’

### Status of the DYZ1 array

**Figure 4:**
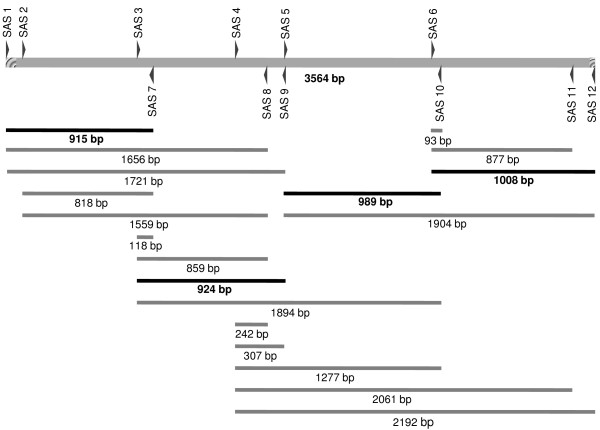
**Diagrammatic illustration showing end point PCR for the amplification of different sub-fractions of the DYZ1 array in DU145 and LNCaP cells.** A single array of DYZ1 is shown on top. Forward and reverse primers are shown above and below the bars. Similarly, amplification products and their corresponding sizes are shown below. The fragments highlighted (dark bar) were used for subsequent cloning and sequencing analysis.

For ascertaining structural status of the DYZ1 array, 6 sets of primers were designed (Table 3A) to amplify 19 fragments (Table 4) from a single 3.56 kb array of DYZ1 (Figure 4). The analysis of all the 19 combinations showed intact DYZ1 arrays both in DU145 and LNCaP.

**Table 4:** **List of *DYZ*1 primer combinations used for end point PCR, their corresponding amplicon sizes and reaction conditions**

**Serial No.**	**DYZ1, Primer Combinations**	**Amplicon Size (bp)**	**PCR conditions (Annealing and Extension)**
**1**	SAS (1&7)	915	64°C-1.0’, 72°C −1.0’
**2**	SAS (1&8)	1656	65°C −1.0’, 72°C −2.0’
**3**	SAS (1&9)	1721	61°C −1.0’, 72°C −2.0’
**4**	SAS (2&7)	818	61°C −1.0’, 72°C −1.0’
**5**	SAS (2&8)	1559	60-°C 1.0’, 72°C −1.5’
**6**	SAS (3&7)	118	55-°C 1.0’, 72°C −1.0’
**7**	SAS (3&8)	859	65°C −1.0’, 72°C −1.0’
**8**	SAS (3&9)	924	61°C −1.0’, 72°C −1.0’
**9**	SAS (3&10)	1894	64-°C 1.0’, 72°C −2.0’
**10**	SAS (4&8)	242	61°C −1.0’, 72°C −1.0’
**11**	SAS (4&9)	307	55°C −1.0’, 72°C −1.0’
**12**	SAS (4&10)	1277	61°C −1.0’, 72°C −1.5’
**13**	SAS (4&11)	2061	62°C −1.0’, 72°C −2.5’
**14**	SAS (4&12)	2192	61°C −1.0’, 72°C −2.5’
**15**	SAS (5&10)	989	61°C −1.0’, 72°C −1.0’
**16**	SAS (5&12)	1904	61°C −1.0’, 72°C −2.0’
**17**	SAS (6&10)	93	61°C −1.0’, 72°C −1.0’
**18**	SAS (6&11)	877	61°C −1.0’, 72°C −1.0’
**19**	SAS (6&12)	1008	64°C −1.0’, 72°C −1.0’

### Copy number calculation of DYZ1 using real time PCR

**Figure 5:**
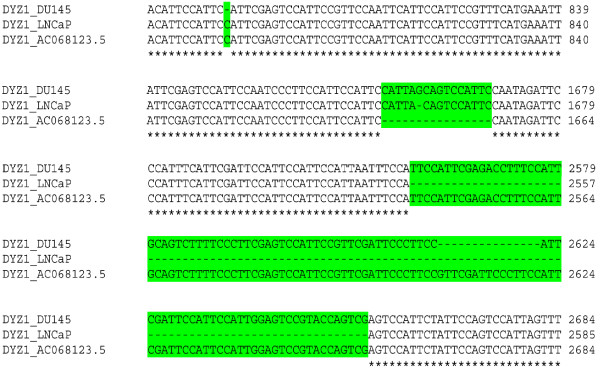
**DYZ1 sequence showing insertion and deletion.** Partial aligned DYZ1 sequence from DU145 and LNCaP showing insertion and deletion compared to that of the reference sequence AC068123.5.

We calculated DYZ1 copy number in DU145 and LNCaP by Real Time PCR using SYBR green chemistry employing absolute quantification method and a standard curve of cloned DYZ1 plasmid using ten-fold dilutions starting with 20 crore (2x10^8^) copies. The amplification plot, dissociation curve and standard curve are given in Figure 5A, B, and C, respectively. Figure 5D represents the number of DYZ1 copies. DU145 contains 742 copies, while LNCaP contains 1945 copies per 3.3 pg of genomic DNA.

### 3564 bp array sequence of DYZ1

**Figure 6:**
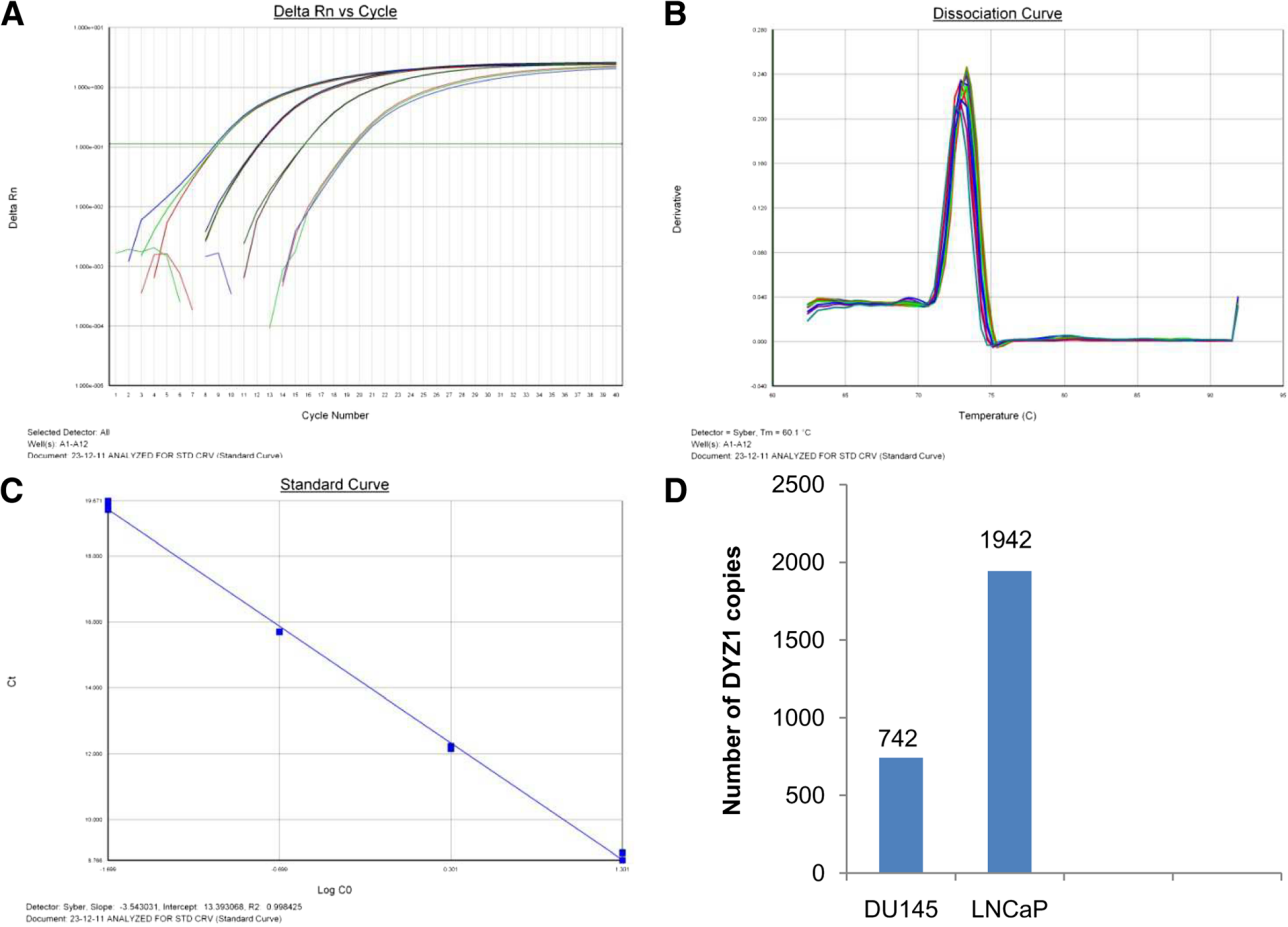
**Copy number estimation of DYZ1 in DU145 and LNCaP.** (**A**) represents the amplification plot, (**B**), the corresponding dissociation curve (**C**), the standard plot and (**D**) shows the number of DYZ1 copies in DU145 and LNCaP.

Four PCR amplified fragments (Figure 4, dark bar) were cloned and sequenced. Sequencing results showed insertion, deletion and several point mutations. Compared to normal sequences of the DYZ1, the number of highly abundant “TTCCA” repeats [25] and its derivatives per 3564 bp (*Hae*III fragment) were found to be different amongst normal, DU145 and LNCaP cells (Table 5 and Additional file 2). The DYZ1 sequences from both DU145 and LNCaP cells were aligned with the reference sequence from NCBI database (Accession no. AC068123.5, Gene ID- 100499443) using ClustalW software (see Additional file 3). Between DU145 and the reference sequence; 23 point mutations, between DU145 and LNCaP; 11 point mutations and between LNCaP and the reference sequence; 27 point mutations were detected. Compared to the reference DYZ1 sequence, DU145 showed Insertion of 16 bp in between the position of 1654^-^ and 1655 bp, deletions of 1 bp at position 792 bp and 15 bp between 2606–2622 position, respectively. LNCaP showed insertion of 15 bp between 1654- 1655 bp position and deletion of 114 bp between 2541–2657 bp. The regions showing major deletions are given in Figure 6 (For complete details, please see Additional file 3). A comparison of LNCaP with DU145 showed a net deletion of 99 bp in LNCaP. Upon virtual restriction mapping of 3.56 Kb unit of DYZ1 using Restriction Mapper version 3 software, loss and gain of several restriction sites were detected (Table 6). Compared to the reference sequence, a total of 6 restriction enzyme sites (*Bsa*BI, *Nla*IV, *Bam*HI, *Eco*RII, *Xho*II and *Csp*CI) were lost and *Eco*57I site was gained in DU145. Similarly, a total 8 restriction enzyme sites (*Bsa*BI, *Nla*IV, *Bam*HI, *Eco*RII, *Xho*II, *Csp*CI, *Mae*II and *Mae*III) were lost and *Eco57*I site was gained in LNCaP. Thus, one *Eco*57I site was gained in both the cell lines. Differences in the restriction site frequency were compared amongst DU145, LNCaP and the reference sequence (Table 6). The result showed DYZ1 arrays are uniquely affected in each category of cells.

**Table 5:** Occurrence of “TTCCA” repeats and its derivatives with single or multiple base alterations per 3564 array of DYZ1 in DU145 and LNCaP with the reference sequence AC068123.5

**Serial No.**	**Repeat unit and derivatives**	**Number of TTCCA repeat units and its derivatives per 3564 bp *Hae *III unit of *DYZ1 *array**		
		**AC068123.5**	**DU145**	**LNCaP**
1	Actual Size (bp)	3564	3564	3465
2	TTCCA	229	235	228
3	1 bp derivatives	292	289	282
4	ATCCA	11	9	9
5	TACCA	3	3	2
6	TTACA	14	13	15
7	TTCAA	21	22	22
8	TTTCA	19	19	19
9	TTCTA	25	25	25
10	TTCCT	32	33	33
11	GTCCA	27	31	28
12	TGCCA	9	10	10
13	TTGCA	25	22	22
14	TTCGA	60	60	57
15	TTCCG	18	17	16
16	CTCCA	14	14	14
17	TCCCA	6	4	4
18	TTCCC	8	7	6
19	2 bp derivatives	142	142	139
20	3 bp derivatives	37	33	30
21	4 bp derivatives	9	9	10
22	5 bp derivatives	1	1	1

**Table 6:** Restriction mapping of 3.56 Kb DYZ1 sequence from DU145, LNCaP and the reference sequence AC068123.5 by virtual digest using Restriction Mapper Version 3 software

**Serial No.**	**Restriction enzyme**	**Sequence**	**Sight Length**	**Overhung**	**Frequency of cut sites**		
					**AC068123.5**	**DU145**	**LNCaP**
1	*Bsa*BI	GATNNNNATC	6	blunt	1	**0**	**0**
2	*Cac*8I	GCNNGC	4	blunt	1	1	1
3	*Nla*IV	GGNNCC	4	blunt	1	**0**	**0**
4	*Bam*HI	GGATCC	6	5’	1	**0**	**0**
5	*Bse*YI	CCCAGC	6	5’	1	1	1
6	*Bsp*1407I	TGTACA	6	5’	1	1	1
7	*Bsp*HI	TCATGA	6	5’	1	1	1
8	*Cla*I	ATCGAT	6	5’	1	1	1
9	*Dde*I	CTNAG	4	5’	1	1	1
10	*Eco*RI	GAATTC	6	5’	1	1	1
11	*Eco*RII	CCWGG	5	5’	1	**0**	**0**
12	*Mae*II	ACGT	4	5’	1	1	**0**
13	*Mae*III	GTNAC	4	5’	1	1	**0**
14	*Vsp*I	ATTAAT	6	5’	1	1	1
15	*Xho*II	RGATCY	6	5’	1	**0**	**0**
16	*Bse*MII	CTCAG	5	3’	1	1	1
17	*Bsg*I	GTGCAG	6	3’	1	1	1
18	*Bts*I	GCAGTG	6	3’	1	1	1
19	*Eco*57I	CTGAAG	6	3’	0	**1**	**1**
20	*Eco*57MI	CTGRAG	6	3’	1	**2**	**2**
21	*Gsu*I	CTGGAG	6	3’	1	1	1
22	*Hpy*188I	TCNGA	4	3’	1	1	1
23	*Mn*lI	CCTC	4	3’	1	1	1
24	*Pfl*MI	CCANNNNNTGG	6	3’	1	1	1
25	*Sdu*I	GDGCHC	6	3’	1	1	1
26	*Scr*FI	CCNGG	4	5’	2	**1**	**1**
27	*Ars*I	GACNNNNNNTTYG	7	3’	2	2	2
28	*Bda*I	TGANNNNNNTCA	6	3’	2	2	2
29	*Csp*CI	CAANNNNNGTGG	7	3’	2	**0**	**0**
30	*Tsp*RI	CASTG	5	3’	2	2	2
31	*Dpn*I	GATC	4	blunt	3	**2**	**2**
32	*Apo*I	RAATTY	6	5’	3	3	3
33	*Asu*II	TTCGAA	6	5’	3	3	3
34	*Eco*31I	GGTCTC	6	5’	3	**2**	**2**
35	*Mae*I	CTAG	4	5’	3	3	**4**
36	*Mbo*I	GATC	4	5’	3	**2**	**2**
37	*Sfa*NI	GCATC	5	5’	3	**4**	**4**
38	*Tat*I	WGTACW	6	5’	3	3	3
39	*Set*I	ASST	4	3’	3	3	**1**
40	*Cvi*JI	RGCY	4	blunt	4	4	4
41	*Msl*I	CAYNNNNRTG	6	blunt	4	4	4
42	*Fok*I	GGATG	5	5’	4	**2**	**2**
43	*Alf*I	GCANNNNNNTGC	6	3’	4	4	4
44	*Rsa*I	GTAC	4	blunt	5	5	**4**
45	*Bcc*I	CCATC	5	5’	5	**3**	**4**
46	*Bsm*AI	GTCTC	5	5’	5	5	**4**
47	*Mse*I	TTAA	4	5’	5	**6**	**6**
48	*Bst*XI	CCANNNNNNTGG	6	3’	5	5	**4**
49	*Mbo*II	GAAGA	5	3’	5	**3**	**3**
50	*Xmn*I	GAANNNNTTC	6	blunt	6	6	6
51	*Tst*I	CACNNNNNNTCC	6	3’	6	**4**	**4**
52	*Nla*III	CATG	4	3’	7	7	7
53	*Bcg*I	CGANNNNNNTGC	6	3’	16	**14**	**14**
54	*Bsr*DI	GCAATG	6	3’	16	**14**	**14**
55	*Bsr*I	ACTGG	5	3’	17	**19**	**18**
56	*Tsp*GWI	ACGGA	5	3’	20	**23**	**17**
57	*Ags*I	TTSAA	5	3’	22	**20**	**23**
58	*Bsm*I	GAATGC	6	3’	22	**23**	**21**
59	*Fai*I	YATR	4	blunt	23	**25**	23
60	*Ple*I	GAGTC	5	5’	23	**21**	**21**
61	*Tsp*DTI	ATGAA	5	3’	25	**24**	**24**
62	*Tsp*EI	AATT	4	5’	35	35	35
63	*Tfi*I	GAWTC	5	5’	52	**51**	**51**
64	*Taq*I	TCGA	4	5’	66	**62**	**62**
65	*HinfI*	GANTC	4	5’	75	**72**	**72**

The loss or gains of the restriction sites with respect to the reference sequence are highlighted in bold.

### FISH analysis

**Figure 7:**
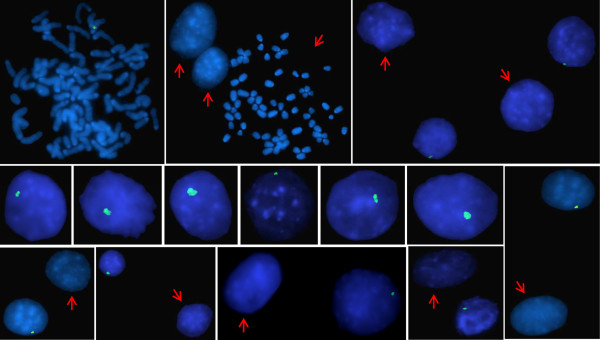
**Localization of DYZ1 in DU145 cells by FISH.** DAPI (4’, 6-diamidino-2-phenylindole) stained metaphases and interphase nuclei are shown having green signal of DYZ1. Nuclei and metaphases lacking DYZ1 are indicated by red arrow. Note the variation in the DYZ1 signal intensities across nuclei reflecting copy number variation.

The prostate cells DU145 might have encountered many changes including translocation of the part of 20th chromosome to the Y chromosome. The DU145 Y chromosome was found to harbour only 742 copies of DYZ1 which is far less than that in a normal Y chromosome. The DYZ1 constitutes approximately 20% of the normal human Y chromosome needed for the maintenance of its structural and functional integrities. A decrease in the number of arrays seems to threaten the survival of Y chromosome in DU145 cell. To ascertain the presence or the absence of Y chromosome in DU145 cells, we conducted FISH experiment using a labelled DYZ1 probe. Approximately, 400 nuclei and metaphases were screened. To rule out the possibility of experimental error, as a positive control, the metaphases from the normal human male were hybridized with DYZ1 probe. Several nuclei and metaphases in DU145 were found to be devoid of DYZ1 fluorescence signals. This way, approximately, 48% of DU145 cells were found to have no Y chromosome (Figure 7).

## Discussion

DU145 and LNCaP cells originated from prostate tumours. Despite involvement of several chromosomes in the etiology of PC, our focus remained on the Y chromosome of the two cell lines to assess several Y linked genes and DYZ1 regions. Sequences of the *SRY* gene were found to be normal in both the cells. Previous studies conducted on PC cell lines employed conventional G-banding, FISH, CGH and SKY approaches. However, these techniques owing to innate limitations, do not detect microdeletions. Despite 51 STSs encompassing *AZFa*, *AZFb* and *AZFc* screened in this study, no microdeletions were detected suggesting that MSY remained intact in both the cell lines.

In an earlier report, Y chromosome was excluded from the CGH analysis because of the presence of a large heterochromatic region [39]. In this study, we particularly focused on the heterochromatic region DYZ1 and assessed not only its copy number variation but also indels all along the length of the array. We detected 742 copies in DU145 and that of 1945 in LNCaP cells. Significant difference in the copy number of DYZ1 between DU145 and LNCaP could be due to the unequal number of Y chromosome(s) per cell. It may be noted that DU145 carries single Y chromosome per cell coupled with a major translocation of part of 20th chromosome to the Y chromosome, whereas LNCaP carries 2 Y chromosomes per cell [3]. In an earlier study on the biopsied samples of PC, we detected 550 copies of the DYZ1 [26] which is in accordance with our present study.

Though MSY was intact in DU145 and LNCaP cells but DYZ1 was clearly affected showing insertion, deletion, frequent point mutations and copy number variations. These mutational events, not only shifts the frame of abundant “TTCCA” repeats but also generate its derivatives leading to the shrinkage or expansion of the same. The insertion and deletion of 16 bp kept the frame unchanged and maintained the original length of array of 3564 bp in DU145. Due to insertion of 15 bp and deletion of 114 bp, the LNCaP cells showed net loss of 99 bp in the array of 3564 bp (Additional file 3). A normal array of 3564 bp harbours approximately 80 different restriction enzyme recognition sites [25]. We detected more loss of restriction sites in both the cell lines than that of their gains. Startlingly, a single *Eco57*I site was gained in both the cell types though its biological significance remained unclear. These results clearly suggest that DYZ1 is indeed affected during the process of cells becoming cancerous. Despite these changes, Y chromosome survived in about 58% of DU145 cells (Figure 7). Most likely, such DU145 cells have managed to retain the critical number of the DYZ1 copies with near normal sequences needed for the sustenance of the Y chromosome.

The African-American men have been found to show highest and Japanese, the lowest incidences of PC in the world [40-42]. This suggests that males from different ethnicity and geographical regions may be different with respect to their susceptibility to develop prostate cancer. It may be noted that status of DYZ1 was not assessed in the males from either of these populations. Based on our study, we presume that besides susceptibility to PC, males from different ethnic and geographical regions may show sequence and copy number variations in the DYZ1 arrays. However, this warrants a detailed analysis of sufficient number of PC males before a conclusion can be drawn. When, once this issue is fully resolved, DYZ1 may be added amongst the list of possible bio-marker for DNA based diagnosis using PC biopsied samples.

## Conclusions

Our study demonstrates that, MSY region and *SRY* gene both remain intact in DU145 and LNCaP PC cell lines. Since, DYZ1 region is maximally affected both in terms of sequence and array’s copy number; this may be exploited as possible bio-marker for DNA based diagnosis of PC together with other marker systems.

## Methods

### Cell culture and DNA isolation

DU145 and LNCaP cell lines were available with National Institute of Immunology, New Delhi and Jawaharlal Nehru University, New Delhi, respectively in connection with other projects. Institute and University procured this cell lines from ATCC (American Type Culture Collection, Manassas, USA). DU145 cells were cultured in T75 flasks containing 10% DMEM (Dulbecco’s Modified Eagle Medium, Life Technologies, Gibco, USA). LNCaP cells were cultured in T75 flask containing 10% RPMI 1640 (Life Technologies, Gibco, USA). Cultures were supplemented with 1% antibiotic and antimycotic solution.

DNA from cultured cells was isolated using DNeasy Blood and Tissue kit (Qiagen, Germany). Quality of isolated DNA was checked by electrophoresis using 1% agarose gel and concentration was measured spectrophotometrically.

### Sequence Tagged Site (STS) mapping of MSY region

MSY region was analysed for micro-deletions using STS end point PCR reactions. A total of 51 STSs were selected from the MSY breakpoint mapper for screening this region. The end point PCR reactions were performed in 20 μl reaction volumes containing 5X Green Go Taq reaction buffer (Promega, Madison, USA), 200 μM dNTPs (Biotools, Spain), 1 IU Go Taq polymerase (Promega, Madison, USA) and 100 ng of genomic DNA. STS PCR primers were procured from Sigma-Aldrich, USA. STS screening was performed following the PCR conditions available in MSY Breakpoint Mapper database [17]. β-actin (Forward primer- 5’ AGATGACCCAGATCATGTTTGAGA 3’ and Reverse primer- 5’ CTAAGTCATAGTCCGCCTAGAAGC) and sY14 (Additional file 4) primers were used as control for assessing the quality of genomic DNA. Genomic DNA from normal males were used as positive controls. Female genomic DNA and a reaction without template were used as negative controls. The amplified products were resolved on 1.5% agarose gel, stained with ethidium bromide and visualized under UV illumination.

### Single Nucleotide Variants (SNV) analysis

The *AZFc* region of MSY was analyzed for 7 SNVs in the *DAZ* region and one SNV for *GOLY1, BPY2* and *TTTY4* by PCR-restriction fragment length polymorphism. The details of primer sequences, accession numbers, product sizes and restriction enzymes used for SNV analysis are given in the Table 2. SNV PCR reactions were performed in 40 μl following standard PCR conditions. The amplified PCR product was precipitated by addition of 4 μl of 3 M sodium acetate and 120 μl of absolute ethanol and incubated at -70°C for 4 hours. DNA was pelleted down at 13,000 rpm for 20 minutes at 4°C, washed in 70% ethanol, dried and dissolved in 10 μl water. The purified PCR products were further subjected to restriction digestion using appropriate restriction enzymes and buffer. The digested fragments were resolved on 2.5% agarose gel, stained with ethidium bromide and visualized under UV illumination.

### Cloning and sequencing of *SRY* and DYZ1 array

Pfu DNA polymerase (Biotools, Spain) amplified amplicons were resolved on 1.5% agarose gel, sliced and eluted using gel extraction kit (Fermentas, Thermo Fischer Scientific). Eluted DNA was cloned in pGEM®-T easy vector (Promega, Madison, USA). 4 recombinant clones for each fragment were sequenced on Applied Biosystems 3130xl Genetic Analyzer using ABI PRISM® BigDye® terminator v3.1 cycle sequencing kit (Life Technologies, California, USA). For cycle sequencing, PCR reactions were set as 96°C for 1 minute, followed by 25 cycles each consisting of 96°C for 10 seconds, 50°C for 5 seconds, and 60°C for 4 minutes. After PCR, extension products were purified using ethanol–sodium acetate precipitation method followed by washing in 70% ethanol. Hi-Di™ Formamide (Life Technologies, California, USA) was added, samples were heat denatured, chilled on ice and loaded on ABI 3130xl genetic analyzer. Sequencing was performed using Run 3130xl Data Collection software v3.0, sequences were retrieved using Sequencing Analysis 5.3.1 and sequence analysis was done using Gene runner software.

### Analysis of DYZ1 array

The intactness of DYZ1 array was assessed using end point PCR. The details of primers, their combinations and PCR conditions are listed in the Table 4. Copy number of DYZ1 was calculated based on absolute quantification method using qPCR. DNA was used as template and SYBR green (Life Technologies, California, USA) was used as detection dye. The qPCR reactions were performed on Sequence Detection System 7500 (Life Technologies, California, USA). 10 fold dilutions of recombinant plasmid containing ~3.56 Kb *Hae*III fragment of DYZ1 array were used to generate standard curve starting with 2 X 10^8^ copies. All the reactions were carried out in triplicates using three different concentrations of the template DNA from DU145 and LNCaP cells. The standard curve had a slope of −3.32 and R^2^ value of >0.99. Copies of the DYZ1 array were calculated by extrapolation of the standard curve obtained with known copies of the recombinant plasmid.

### Florescence *in-situ* hybridization (FISH)

DU145 cells cultured in 10% DMEM were used for metaphase chromosome preparation. These cells were grown for 70 hours in 5% CO_2_ environment at 37°C and then treated with colcemid (3 μg/ml). Treated cells were again incubated for 2 hours in 5% CO_2_ environment at 37°C. After 72 hours, cells were centrifuged at 1800 rpm for 10 minutes at room temperature (RT). Harvested cells were resuspended in 0.075 M KCl and incubated at RT for 20 minutes in 5% CO_2_ environment at 37°C. Then added 1 drop of fixative solution (3:1, methanol: glacial acetic acid) and centrifuged at 1800 rpm for 10 minutes at RT. Discarded the supernatant, resuspended the cell pellet in 10 ml fresh fixative solution and incubated for 20 minutes at 37°C. Then centrifuged cells at 1800 rpm for 10 minutes at RT. Repeated the washing step 2 times. Finally, cells were resuspended in fresh 1 ml fixative and stored at -20°C.

For metaphase chromosome preparation, 20 μl of nuclei suspension in fixative was spread on the glass slides. Before proceeding further, slides were kept for 1 week at 37°C for ageing. Slides were then incubated in 70% glacial acetic acid for 2 minutes followed by dehydration in 70%, 90% and 100% ethanol for 2 minutes each at RT. Air dried the glass slides and incubated in a solution containing 0.1 mg/ml and 0.01 N HCl for 20 minutes. Fixed the metaphase preparation in 4% paraformaldehyde (prepared in 1X PBS, pH 7.4) for 5 minutes at RT. Slides were then washed in 1.0 M Tris–HCl (pH 7.4) followed by 2 washes in 1X phosphate buffer saline (PBS) for 5 minutes each at RT. Further, incubated in 0.5% Triton-X-100 (prepared in 1X PBS) for 10 minutes followed by 3 washes in 1X PBS for 5 minutes each. Slides were then incubated in 0.1 N HCl for 10 minutes followed by 3 washes in 1X PBS for 5 minutes each and stored in 2X SSC (pH 7.4) overnight at 4°C until used for hybridization. FISH was conducted with a labelled clone containing 3.56 kb sequence of DYZ1. Labelling was done with biotin-dUTP using a Nick translation kit from Vysis (Illinois, USA). Hybridization, washing, counterstaining and mounting of the slides were conducted following standard protocols [43]. The slides were screened under the Olympus fluorescence microscope (BX 51) fitted with vertical fluorescence illuminator U-LH100HG UV, excitation and barrier filters and images were captured with a charge-coupled device (CCD) camera. Captured images were analyzed using CytoVision software version 3.93 from Applied Imaging Systems.

## Abbreviations

PC: Prostate cancer; STS: Sequence tagged site; NRY: Non recombining region of Y; MSY: Male specific region of Y; FISH: Fluorescence *in situ* hybridization; CGH: Comparative genomic hybridization; SKY: Spectral karyotyping; SNV: Single nucleotide variants; DNA: Deoxyribonucleic acid; UV: Ultraviolet; PCR: Polymerase chain reaction; qPCR: Quantitative polymerase chain reaction.

## Competing interests

The authors declare that they have no competing interests.

## Authors’ contribution

SKY and AK carried out the experiments. SKY did *in-silico* analysis, interpreted the data and wrote the manuscript. SA conceived the study, interpreted the results, revised the manuscript critically and provided overall supervision. All the authors read and approved the final manuscript.

## Supplementary Material

Additional file 1 **Multiple Sequence Alignment (MSA) of the *SRY *gene cloned and sequenced from DU145 and LNCaP with that of normal sequence (Accession number- NM_003140) in the database.** Coding region starts at 89^th^bp and ends at 703 bp. MSA showed no sequence alteration within the *SRY *gene. Click here for file

Additional file 2 Complete 3.56 Kb DYZ1 sequences of the reference sequence AC068123.5 (A), DU145 (B), and LNCaP (C), all showing in frame arrangement of the pentanucleotide motifs. Click here for file

Additional file 3 **Multiple Sequence Alignment of 3.56 Kb sequence of *DYZ1 *array from DU145 and LNCaP with sequence from the NCBI database (Accession no. AC068123.5, Gene ID- 100499443). Perfectly aligned sequences are indicated by star and deletions, by hyphens. Mismatched base pairs, deletions or insertions are highlighted in green colour.** Click here for file

Additional file 4 Details of STS primers used for screening MSY region. Click here for file
