# Supplementary material for: Fate of the human Y chromosome linked genes and loci in prostate cancer cell lines DU145 and LNCaP
Source: BMC Genomics. 2013 May 11;14:323. doi: 10.1186/1471-2164-14-323 (PMC3660188; doi:10.1186/1471-2164-14-323)
Supplement: Additional file 1 — Multiple Sequence Alignment (MSA) of the SRY gene cloned and sequenced from DU145 and LNCaP with that of normal sequence (Accession number- NM_003140) in the database. Coding region starts at 89thbp and ends at 703 bp. MSA showed no sequence alteration within the SRY gene. [file 1471-2164-14-323-S1.pdf]

Additional file 2: Multiple Sequence Alignment (MSA) of the *SRY* gene cloned and sequenced from DU145 and LNCaP with that of normal sequence (Accession number- NM_003140) in the database. Coding region starts at 89th bp and ends at 703 bp. MSA showed no sequence alteration within the *SRY* gene.

```
SRY_NM_003140        GAATCTGGTAGAAGTGAGTTTTGGATAGTAAAATAAGTTTCGAACTCTGGCACCTTTCAA 60
SRY_LNCaP            GAATCTGGTAGAAGTGAGTTTTGGATAGTAAAATAAGTTTCGAACTCTGGCACCTTTCAA 60
SRY_DU145            GAATCTGGTAGAAGTGAGTTTTGGATAGTAAAATAAGTTTCGAACTCTGGCACCTTTCAA 60
                     ************************************************************

SRY_NM_003140        TTTTGTCGCACTCTCCTTGTTTTTGACAATGCAATCATATGCTTCTGCTATGTTAAGCGT 120
SRY_LNCaP            TTTTGTCGCACTCTCCTTGTTTTTGACAATGCAATCATATGCTTCTGCTATGTTAAGCGT 120
SRY_DU145            TTTTGTCGCACTCTCCTTGTTTTTGACAATGCAATCATATGCTTCTGCTATGTTAAGCGT 120
                     ************************************************************

SRY_NM_003140        ATTCAACAGCGATGATTACAGTCCAGCTGTGCAAGAGAATATTCCCGCTCTCCGGAGAAG 180
SRY_LNCaP            ATTCAACAGCGATGATTACAGTCCAGCTGTGCAAGAGAATATTCCCGCTCTCCGGAGAAG 180
SRY_DU145            ATTCAACAGCGATGATTACAGTCCAGCTGTGCAAGAGAATATTCCCGCTCTCCGGAGAAG 180
                     ************************************************************

SRY_NM_003140        CTCTTCCTTCCTTTGCACTGAAAGCTGTAACTCTAAGTATCAGTGTGAAACGGGAGAAAA 240
SRY_LNCaP            CTCTTCCTTCCTTTGCACTGAAAGCTGTAACTCTAAGTATCAGTGTGAAACGGGAGAAAA 240
SRY_DU145            CTCTTCCTTCCTTTGCACTGAAAGCTGTAACTCTAAGTATCAGTGTGAAACGGGAGAAAA 240
                     ************************************************************

SRY_NM_003140        CAGTAAAGGCAACGTCCAGGATAGAGTGAAGCGACCCATGAACGCATTCATCGTGTGGTC 300
SRY_LNCaP            CAGTAAAGGCAACGTCCAGGATAGAGTGAAGCGACCCATGAACGCATTCATCGTGTGGTC 300
SRY_DU145            CAGTAAAGGCAACGTCCAGGATAGAGTGAAGCGACCCATGAACGCATTCATCGTGTGGTC 300
                     ************************************************************

SRY_NM_003140        TCGCGATCAGAGGCGCAAGATGGCTCTAGAGAATCCCAGAATGCGAAACTCAGAGATCAG 360
SRY_LNCaP            TCGCGATCAGAGGCGCAAGATGGCTCTAGAGAATCCCAGAATGCGAAACTCAGAGATCAG 360
SRY_DU145            TCGCGATCAGAGGCGCAAGATGGCTCTAGAGAATCCCAGAATGCGAAACTCAGAGATCAG 360
                     ************************************************************

SRY_NM_003140        CAAGCAGCTGGGATACCAGTGGAAAATGCTTACTGAAGCCGAAAAATGGCCATTCTTCCA 420
SRY_LNCaP            CAAGCAGCTGGGATACCAGTGGAAAATGCTTACTGAAGCCGAAAAATGGCCATTCTTCCA 420
SRY_DU145            CAAGCAGCTGGGATACCAGTGGAAAATGCTTACTGAAGCCGAAAAATGGCCATTCTTCCA 420
                     ************************************************************

SRY_NM_003140        GGAGGCACAGAAATTACAGGCCATGCACAGAGAGAAATACCCGAATTATAAGTATCGACC 480
SRY_LNCaP            GGAGGCACAGAAATTACAGGCCATGCACAGAGAGAAATACCCGAATTATAAGTATCGACC 480
SRY_DU145            GGAGGCACAGAAATTACAGGCCATGCACAGAGAGAAATACCCGAATTATAAGTATCGACC 480
                     ************************************************************

SRY_NM_003140        TCGTCGGAAGGCGAAGATGCTGCCGAAGAATTGCAGTTTGCTTCCCGCAGATCCCGCTTC 540
SRY_LNCaP            TCGTCGGAAGGCGAAGATGCTGCCGAAGAATTGCAGTTTGCTTCCCGCAGATCCCGCTTC 540
SRY_DU145            TCGTCGGAAGGCGAAGATGCTGCCGAAGAATTGCAGTTTGCTTCCCGCAGATCCCGCTTC 540
                     ************************************************************

SRY_NM_003140        GGTACTCTGCAGCGAAGTGCAACTGGACAACAGGTTGTACAGGGATGACTGTACGAAAGC 600
SRY_LNCaP            GGTACTCTGCAGCGAAGTGCAACTGGACAACAGGTTGTACAGGGATGACTGTACGAAAGC 600
SRY_DU145            GGTACTCTGCAGCGAAGTGCAACTGGACAACAGGTTGTACAGGGATGACTGTACGAAAGC 600
                     ************************************************************
```

```
SRY_NM_003140        CACACACTCAAGAATGGAGCACCAGCTAGGCCACTTACCGCCCATCAACGCAGCCAGCTC 660
SRY_LNCaP            CACACACTCAAGAATGGAGCACCAGCTAGGCCACTTACCGCCCATCAACGCAGCCAGCTC 660
SRY_DU145            CACACACTCAAGAATGGAGCACCAGCTAGGCCACTTACCGCCCATCAACGCAGCCAGCTC 660
                     ************************************************************

SRY_NM_003140        ACCGCAGCAACGGGACCGCTACAGCCACTGGACAAAGCTGTAGGACAATCGGGTAACATT 720
SRY_LNCaP            ACCGCAGCAACGGGACCGCTACAGCCACTGGACAAAGCTGTAGGACAATCGGGTAACATT 720
SRY_DU145            ACCGCAGCAACGGGACCGCTACAGCCACTGGACAAAGCTGTAGGACAATCGGGTAACATT 720
                     ************************************************************

SRY_NM_003140        GGCTACAAAGACCTACCTAGATGCTCCTTTTTACGATAACTTACAGCCCTCACTTTCTTA 780
SRY_LNCaP            GGCTACAAAGACCTACCTAGATGCTCCTTTTTACGATAACTTACAGCCCTCACTTTCTTA 780
SRY_DU145            GGCTACAAAGACCTACCTAGATGCTCCTTTTTACGATAACTTACAGCCCTCACTTTCTTA 780
                     ************************************************************

SRY_NM_003140        TGTTTAGTTTCAATATTGTTTTCTTTTCTCTGGCTAATAAAGGC 824
SRY_LNCaP            TGTTTAGTTTCAATATTGTTTTCTTTTCTCTGGCTAATAAAGGC 824
SRY_DU145            TGTTTAGTTTCAATATTGTTTTCTTTTCTCTGGCTAATAAAGGC 824
                     ********************************************
```
