# Supplementary material for: Fate of the human Y chromosome linked genes and loci in prostate cancer cell lines DU145 and LNCaP
Source: BMC Genomics. 2013 May 11;14:323. doi: 10.1186/1471-2164-14-323 (PMC3660188; doi:10.1186/1471-2164-14-323)
Supplement: Additional file 2 — Complete 3.56 Kb DYZ1 sequences of the reference sequence AC068123.5 (A), DU145 (B), and LNCaP (C), all showing in frame arrangement of the pentanucleotide motifs. [file 1471-2164-14-323-S2.pdf]

Additional file 3: Complete 3.56 Kb DYZ1 sequence of the reference sequence AC068123.5 (A), DU145 (B), and LNCaP(C), all showing in frame arrangement of the pentanucleotide motifs.

(A) DYZ1 reference sequence_AC068123.5

```
--CCT GTCCA TTACA CTACA TTCCC TTCCA TTCCA ATGAA TTCCA TTCCA TTCCA ATCCA TTCCT TTCCT 68
TTCGC TTGCA TTCCA TTCTA TTCTC TTCTA CTGCA TACAA TTTCA CTCCA TTCGT TCCCA TTCCA TTCAA 138
TTCCA TTCCA TTCAA TTCCA TTCCA TTTGT TTCCA TTCTC TTCGA TTCCA TTTCT TTATA TTCCA TGCCA 208
TTCGA TTCCA TTCTA TTGGG TTGCA TTACA TTCGT GTTCA TTCCA TTCCA GACCA TTCCA TTTGA CTCCA 278
TTCCT TTCGA GCCCT TTCAA TTTGA GTCCA TTCCT TTCCA GTCCA TTTCA CTCCA GTCCA TTACT ATCCA 348
TTCCA TACCA TTCCA TCCCA TTCCA TTCCA TTCCA TTCCA TTCCA TTCCA TTCCA TTCCA TTGCA TTCCA 418
TTCCA TTCCA TTCCA TTGCA CTGCA CTCCA TTCCA TTACA TTCTA CTCTA TCTGA GTCGA TTTTA TTGCA 488
TTAGA TTCTA TTCCA TTGGA TTACT TTCCA TTCGA TTACA TTCCA TTCAT GTACA TTCCA TTCCA GTCAA 558
TTACA TTCGA GTTCA TTACG TTACA TTCCT GTATA TTCCA TTGTA TTCGA TCCCA TTCCT TTCAA TTCCA 628
TTTCA TTCGA CTCCA TTATA TTCAA TTCCA TTCCA CTCGA ATCCA TTCCA TTAGA GGACA TTCCA TTCCA 698
ATGCA TTCCA TTCCA TTCCA TAGCA TTCCA TTGCA TTCGA TTCCA TTCCA TTTGA TGTCA TTCCA TTTGA 768
TGCCA TCCCA TGACA TTCCA TTCCA TTCGA GTCCA TTCCG TTCCA ATTCA TTCCA TTCCG TTTCA TGAAA 838
TTCGA GTCCT TTCCA GTACA TTTCA TTCCA ATCCC ATCCA ATCCA ATCTA CTCCA TTCAA TTCCT TTCCA 908
TTCCA TTTGA TTTGA TTCCA TTGAT TTG-A TTCCA TTCAG TTTGA TTCCA TTCCG TGAAA TTTCG TTCCA 977
TTCTA TTCCA TTGCA TTACT TTCCA TTCAA TTCCA TTCCA TTTCA TTTCA GTCCA TTCGC TTCCT TTCCT 1047
TTCGA TTCAA TTCCA TTTGA TTCCA CTCCA TTCTA TGCGA TTTCA TTCCA ATCGA TTCAA TTCCA TTCGA 1117
TGACA TTCCT TTCGT TTCCA TTCCA TTCGA GTCCA TTTAA TTTGA --GCA TTCGT GTCCA TTCTA TTCGA 1185
GTCCA TTCCA TTACA GTCTA TTCTA TTCCC TTCCA TTCGT GTTGA TTCAA TTTCA TTCCC TTCCA TTCGA 1255
TTCCT TTCCA TTCGA TTCCA TTCCT TTCCA TTCCA TTCCA TTCGT TCCCA TTCCA TGTGA TTTCA TTCCA 1325
TTCCA GTCCA TTATA TTCGA GTCCA CTCCA CTCCA TTCTA TTACA TTCAA TTCCT TTTGA GTCCG TTCCA 1395
TAACA CTCCA TTCAT TTCGA TTCCA TTTCT TGCCA GTTTT ---C- TTCCA TTTTA TTCCA TTCCG TTCGA 1461
TTCCA TTCCA TTCGA TTGCA TTCCA TTCGA ATCCT TTCCA TTCCA TTTCA TTCCA TTCCT TTCTA TTCCA 1531
TTCCA TTTCA TTCGA TTTGA TTCCA TTCTG TTCTA TTCCA TTCAA TTCTT TTTCA TTCCA TTCGA ATCCT 1601
TTCTA TTGCA GTCCA TTCCA TTCGA GTCCA TTCCA ATCCC TTCCA TTCCA TTCCA ATAGA TTCCA TTCCT 1671
TTGCC TTCCA TTCGA ATCCA TTCCA TTCTA GTCCA TTCCA TTTGA GTCAA TTCCA TTCCA TTCCA TTCTA 1741
TTCCT TTCCA ATCCA TTCGA TTCCA TTCGA TTCAA TTCCA TTTGA TTCTC TTTCA TTCTA TTTTA TTCCA 1811
TGCCA TTTGA TTGCA TTGCA TTCCA TTCCG TTTGA TTCCA ATCCA TCCAA GAAAG TTCCA TTCCA GTCCA 1881
TTGCT TTCCA GTCCA TTCCA TTCCA CTCTA GTCTA TTCCA CTCCA TTCCC TTCCA TTGCA TTCCA TACTA 1951
TTCCA TTCCA TTCCT TTGCA TTCCG ----T TTCCA ATCTC TTCGA GTCCA TTGCA TTCCA GTCCA ATCCA 2017
TTCGA TTACA TTCCT TTTGA TTCCC TGCCA GTCGA TTGCA TTGCA TACTA CACCA TTCCA AAGGA GTTCA 2087
TTCCA TTCTA TCTCC ACACT TTCCA TTCCA CTCTG TTTGA GTCCA TTCCA TTCCA GTCCA TTTAA TTCAA 2157
GGGCA TTCCA TTCCA TTCCA TTCCA TTCCA TTTCA TATTA TTCCA TTCCA TTCAA TTCCA TTCCA GATGA 2227
TTCCA TTCCA TTCTA TACCA TTGCT CTCTG TTCCA TTCCA TTCCA TCTGT CTCCA TTCCT TTCGT TTCGA 2297
TTCCT TTCCA TTCCA TTCCA TTACA TTTGA TCCTA TTTTA TCAAA TTGCA TTCTA TTCGA GTGAT TTCCA 2367
TTCGA GTCCT TTCCA TTCGA TTCCA TTCCA TTCTA TTCCA TTCCT TTGGA TCCCA TTCCA TTCCG TTCCG 2437
TTCAC ATCAA TTCCT GGCGA TTCCA TTACA TTCGA TTTCT TGCCA TTCGA TTCCA TTCCT TTTGA CTCCA 2507
TTTCA TTCGA TTCCA TTCCA TTCCA TTAAT TTCCA TTCCA TTCGA GACCT TTCCA TTGCA GTCTT TTCCC 2577
TTCGA GTCCA TTCCG TTCGA TTCCC TTCCG TTCGA TTCCC TTCCA TTCGA TTCCA TTCCA TTGGA GTCCG 2647
TACCA GTCGA GTCCA TTCTA TTCCA GTCCA TTAGT TTCGA CTCCA TTGCA TTCGA GTGCA TTCCA TTCCG 2717
TGGCT GTCCA TTCCA TTCCG TTTGA TGCCA TTCCA TACGA TTCCA TTCAA TTCGA GACCA TTCTA TTCCT 2787
GTCCA TTCCT TGTGG TTCGA TTCCA TTTCA CTCTA GTCCA TTCCA TTCCA TTCAA TTCCA TTCGA CTCTA 2857
TTCCG TTCCA TTCAA TTCCA TTCCA TTCGA TTCCA TTATT TTCGA GAACC TTGCA TTACA CTCCC TTCCA 2927
TTCCA GTGCA TTCCA TTCCA GTCTC TTCAC TTCGA TTCCA TTCCA TTCGT TTCGA TTCCT TTCCA TTCCA 2997
GCCCA TTCCA TTCCA TTCCA TTCCT TTCCT TTCCG TTTCA TTAGA TTCCA TTGCA TTCGA TTCCA TTCAA 3067
TTCAA TTCCG TGCTA TTCAA TTTGA TTCAT TTCCA TTTAA TTCCA TTCCA TTAGA TTCCA TTCCG TACGA 3137
TTCCA TTCCT TTTGA ATCCA TTCCA TTGGA GTCCA TTCAC TTCCA GAACA TTCCA TTCCA GTCGA ATCCA 3207
TTCGA GTACA TTCCA TTAAA GTTCA TTACA TTCTA ATACA TTCCA TTCCA TTGCA TTCCA TTCCA TTCCA 3277
TTCGA TGCCA TTCGA TTCCA TTCCA TGCCA AATCA TTGCA TTCCT TTCCA TTCCG TTCCT ATCAA TTCCA 3347
TTCCA TTCGA TTTAG TTCGA TTCTA TTCAC TTCCA TTCCA TTCGA TTCCA TTCCA TTGGA GTCAA TTCCT 3417
TTCGA CACCC AGCCT TTCCA GTCAA TGATT TTGGA TTCCA TTTTT TTGCA TTCCA TTACA TTCTA TGACA 3487
TTCGA TTCCG TTTCA TTGCA TTCCA TTCCA TACAT TTTTA TTCCA TTCGA GACCG TAGCA TTCCA CTTTA 3557
TTCCA GG---                                                                   3564
```

(B) DYZ1_DU145

```
--CCT GTCCA TTACA CTACA TTCCC TTCCA TTCCA ATGAA TTCCA TTCCA TTCCA ATCCA TTCCT TTCCT 68
TTCGC TTGCA TTCCA TTCTA TTCCC TTCTA CTGCA TACAA TTTCA CTCCA TTCGT TCCCA TTCCA TTCAA 138
TTCCA TTCCA TTCAA TTCCA TTCCA TTTGT TTCCA TTCTC TTCGA TTCCA TTTCT TTATA TTCCA TGCCA 208
TTCGA TTCCA TTCTA TTGGG TTGCA TTACA TTCGT GTTCA TTCCA TTCCA GACCA TTCCA TTTGA CTCCA 278
TTCCT TTCGA GCCCT TTCAA TTTGA GTCCA TTCCT TTCCA GTCCA TTTCA CTCCA GTCCA TTACT ATCCA 348
TTCCA TACCA TTCCA TCCCA TTCCA TTCCA TTCCA TTCCA TTCCA TTCCA TTCCA TTCCA TTCCA TTCCA 418
TTCCA TTCCA TTCCA TTGCA CTGCA CTCCA TTCCA TTACA TTCTA CTCTA TCTGA GTCGA TTTTA TTGCA 488
TTAGA TTCTA TTCCA TTGGA TTACT TTCCA TTCGA TTACA TTCCA TTCAT GTACA TTCCA TTCCA GTCAA 558
TTACA TTCGA GTTCA TTACG TTACA TTCCA GTATA TTCCA TTGTA TTCGA TCCCA TTCCT TTCAA TTCCA 628
TTTCA TTCGA CTCCA TTATA TTCAA TTCCA TTCCA CTCGA ATCCA TTCCA TTAGA GGACA TTCCA TTCCA 698
ATGCA TTCCA TTCCA TTCCA TAGCA TTCCA TTGCA TTCGA TTCCA TTCCA TTTGA TGCCA TTCCA TTTGA 768
TGCCA TTCCA TGACA TTCCA TTC-A TTCGA GTCCA TTCCG TTCCA ATTCA TTCCA TTCCG TTTCA TGAAA 837
TTCGA GTCCT TTCCA GTACA TTTCA TTCCA ATCCC ATCCA ATCCA ATCTA CTCCA TTCAA TTCCT TTCCA 907
TTCCA TTTGA TTTGA TTCCA TTGAT TTG-A TTCCA TTCAG TTTGA TTCCA TTCCG TGAAA TTTCG TTCCA 976
TTCTA TTCCA TTGCA TTACT TTCCA TTCAA TTCCA TTCCA TTTCA TTTCA GTCCA TTCGC TTCCT TTCCT 1046
TTCGA TTCAA TTCCA TTTGA TTCCA CTCCA TTCTA TGCGA TTTCA TTCCA ATCGA TTCAA TTCCA TTCGA 1116
TGACA TTCCT TTCGT TTCCA TTCCA TTCGA GTCCA TTTAA TTTGA –-GCA TTCGT GTCCA TTCTA TTCGA 1184
GTCCA TTCCA TTACC GTCTA TTCTA TTCCC TTCCA TTCCT GTTGA TTCAA TTTCA TTCCC TTCCA TTCGA 1254
TTCCT TTCCA TTCGA TTCCA TTCCT TTCCA TTCCA TTCCA TTCGT TCCCA TTCCA TGTGA TTTCA TTCCA 1324
TTCCA GTCCA TTATA TTCGA GTCCA CTCCA CTCCA TTCTA TTACA TTCAA TTCCT TTTGA GTCCG TTCCA 1394
TAACA CTCCA TTCAT TTCGA TTCCA TTTCT TGCCA GTTTT –-C-- TTCCA TTTTA TTCCA TTCCG TTCGA 1460
TTCCA TTCCA TTCGA TTGCA TTCCA TTCGA ATCCT TTCCA TTCCA TTTCA TTCCA TTCCT TTCTA TTCCA 1530
TTCCA TTTCA TTCGA TTTGA TTCCA TTCTG TTCTA TTCCA TTCAA TTCTT TTTCA TTCCA TTCGA ATCCT 1600
TTCTA TTGCA GTCCA TTCCA TTCGA GTCCA TTCCA ATCCC TTCCA TTCCA TTCCA T---- TAGCA GTCCA 1666
TTCCA ATAGA TTCCA TTCCT TTGCC TTCCA TTCGA ATCCA TTCCA TTCTA GTCCA TTCCA TTTGA GTCAA 1736
TTCCA TTCCA TTCCA TTCTA TTCCT TTCCA ATCCA TTCGA TTCCA TTCGA TTCAA TTCCA TTTGA TTCTC 1806
TTTCA TTCTA TTTTA TTCCA TGCCA TTTGA TTGCA TTGCA TTCCA TTCCG TTTGA TTCCA GTCCA TTCAA 1876
GAAAG TTCCA TTCCA GTCCA TTGCT TTCGA GTCCA TTCCA TTCCA CTCTA GTCTA TTCCA CTCCA TTCCT 1946
TTCCA TTCCA TTCCA TACTA TTCCA TTCCA TTCCT TTGCA TTCCG T---- TTCCA ATCTA TTCGA GTCCA 2012
TTGCA TTCCA GTCCA GTCCA TTCCA TTACA TTCCT TTTGA TTCCC TGCCA GTCGA TTGCA TTGCA TACTA 2082
CACCA TTCCA AAGGA GTCCA TTCCA TTCTA TCTCA ACACT TTCCA TTCCA CTCTG TTCGA GTCCA TTCCA 2152
TTCCA GTCCA TTTAA TTCAA GGGCA TTCCA TTCCA TTCCA TTCCA TTCCA TTTCA TATTA TTCCA TTCCA 2222
TTCAA TTCCA TTCCA GATGA TTCCA TTCCA TTCTA TACCA TTGCT CTCTG TTCCA TTCCA TTCCA TCTGT 2292
CTCCA TTCCT TTCGT TTCGA TTCCT TTCCA TTCCA TTCCA TTACA TTTGA TCCTA TTTTA TTAAA TTGCA 2362
TTCTA TTCGA GTGAT TTCCA TTCGA GTCCT TTCCA TTCGA TTCCA TTCCA TTCTA TTCCA TTCCT TTGGA 2432
TTCCA TTCCA TTCCG TTCCG TTCAC ATCAA TTCCT TGCGA TTCCA TTACA TTCGA TTTCT TGCCA TTCGA 2502
TTCCA TTCCT TTTGA CTCCA TTTCA TTCGA TTCCA TTCCA TTCCA TTAAT TTCCA TTCCA TTCGA GACCT 2572
TTCCA TTGCA GTCTT TTCCC TTCGA GTCCA TTCCG TTCGA TTCCC TTCCA TTCGA TTCCA TTCCA TTGGA 2642
GTCCG TACCA GTCGA GTCCA TTCTA TTCCA GTCCA TTAGT TTCGA CTCCA TTGCA TTCGA GTGCA TTCCA 2712
TTCCG TGGCT GTCCA TTCCA TTCCG TTTGA TGCCA TTCCA TACGA TTCCA TTCAA TTCGA GACCA TTCTA 2782
TTCCT GTCCA TTCCT TGTGG TTCGA TTCCA TTTCA CTCTA GTCCA TTCCA TTCCA TTCAA TTCCA TTCGA 2852
CTCTA TTCCG TTCCA TTCAA TTCCA TTCCA TTCGA TTCCA TTTTT TTCGA GAACC TTCCA TTACA CTCCC 2922
TTCCA TTCCA GTGCA TTCCA TTCCA GTCTC TTCAG TTCGA TTCCA TTCCA TTCGT TTCGA TTCCT TTCCA 2992
TTCCA GCCCA TTCCA TTCCA TTCCA TTCCT TTCCT TTCCG TTTCA TTAGA TTCCA TTGCA TTCGA TTCCA 3062
TTCAA TTCAA TTCCG TGCTA TTCAA TTTGA TTCAT TTCCA TTTAA TTCCA TTCCA TTAGA TTCCA TTCCG 3132
TACGA TTCCA TTCCT TTTGA ATCCA TTCCA TTGGA GTCCA TTCAC TTCCA GAACA TTCCA TTCCA GTCGA 3202
ATCCA TTCGA GTACA TTCCA TTAAA GTTCA TTACA TTCTA ATACA TTCCA TTCCA TTGCA TTCCA TTCCA 3272
TTCCA TTCGA TGCCA TTCGA TTCCA TTCCA TGCCA AATCA TTGCA TTCCT TTCCA TTCCG TTCCT ATCAA 3342
TTCCA TTCCA TTCGA TTTAG TTCGA TTCTA TTCAC TTCCA TTCCA TTCGA TTCCA TTCCA TTGGA GTCAA 3412
TTCCT TTCGA CACCC AGCCT TTCCA GTCAA TGATT TTGGA TTCCA TTTTT TTGCA TTCCA TTACA TTCTA 3482
TGACA TTCGA TTCCG TTTCA TTGCA TTCCA TTCCA TACAT TTTTA TTCCA TTCGA GACCG TAGCA TTCCA 3552
CTTTA TTCCA GG---                                                                 3564
```

(C) DYZ1_LNCaP

```
--CCT GTCCA TTACA CTACA TTCCC TTCCA TTCCA ATGAA TTCCA TTCCA TTCCA ATCCA TTCCT TTCCT 68
TTCGC TTGCA TTCCA TTCTA TTCCC TTCTA CTGCA TACAA TTTCA CTCCA TTCGT TCCCA TTCCA TTCAA 138
TTCCA TTCCA TTCAA TTCCA TTCCA TTTGT TTCCA TTCTC TTCGA TTCCA TTTCT TTATA TTCCA TGCCA 208
TTCGA TTCCA TTCTA TTGGA TTGCA TTACA TTCGT GTTCA TTCCA TTCCA GACCA TTCCA TTTGA CTCCA 278
TTCCT TTCGA GCCCT TTCAA TTTGA GTCCA TTCCT TTCCA GTCCA TTTCA CTCCA GTCCA TTACT ATCCA 348
TTCCA TACCA TTCCA TCCCA TTCCA TTCCA TTCCA TTCCA TTCCA TTCCA TTCCA TTCCA TTGCA TTCAA 418
TTCCA TTCCC TTCCA TTGCA CTGCA CTCCA TTCCA TTACA TTCTA CTCTA TCTGA GTCGA TTTTA TTGCA 488
TTAGA TTCTA TTCCA TTGGA TTACT TTCCA TTCGA TTACA TTCCA TTCAT GTACA TTCCA TTCCA GTCAA 558
TTACA TTCGA GTTCA TTACA TTACA TTCCA GTATA TTCCA TTGTA TTCGA TCCCA TTCCT TTCAA TTCCA 628
TTTCA TTCGA CTCCA TTATA TTCGA TTCCA TTCCA CTCGA ATCCA TTCCA TTAGA GGACA TTCCA TTCCA 698
ATGCA TTCCA TTCCA TTCCA TAGCA TTCCA TTGCA TTCGA TTCCA TTCCA TTTGA TGCCA TTCCA TTTGA 768
TGCCA TTCCA TGACA TTCCA TTCCA TTCGA GTCCA TTCCG TTCCA ATTCA TTCCA TTCCG TTTCA TGAAA 838
TTCGA GTCCT TTCCA GTACA TTTCA TTCCA ATCCC ATCCA ATCCC ATCTA CTCCA TTCAA TTCCT TTCCA 908
TTCCA TTTGA TTTGA TTCCA TTGAT TT-GA TTCCA TTCAG TTTGA TTCCA TTCCG TGAAA TTTCG TTCCA 977
TTCTA TTCCA TTGCA TTACT TTCCA TTCAA TTCCA TTCCA TTTCA TTTCA GTCCA TTCGC TTCCT TTCCT 1047
TTCGA TTCAA TTCCA TTTGA TTCCA CTCCA TTCTA TGCGA TTTCA TTCCA ATCGA TTCAA TTCCA TTCGA 1117
TGACA TTCCT TTCGT TTCCA TTCCA TTCGA GTCCA TTTAA TTTGA –-GCA TTCGT GTCCA TTCTA TTCGA 1185
GTCCA TTCCA TTACC GTCTA TTCTA TTCCC TTCCA TTCCT GTTGA TTCAA TTTCA TTCCC TTCCA TTCGA 1255
TTCCT TTCCA TTCGA TTCCA TTCCT TTCCA TTCCA TTCCA TTCGT TCCCA TTCCA TGTGA TTTCA TTCCA 1325
TTCCA GTCCA TTATA TTCGA GTCCA CTCCA CTCCA TTCTA TTACA TTCAA TTCCT TTTGA GTCCG TTCCA 1395
TAACA CTCCA TTCAT TTCGA TTCCA TTTCT TGCCA GTTTT –-C-- TTCCA TTTTA TTCCA TTCCG TTCGA 1461
TTCCA TTCCA TTCGA TTGCA TTCCA TTCGA ATCCT TTCCA TTCCA TTTCA TTCCA TTCCT TTCTA TTCCA 1531
TTCCA TTTCA TTCGA TTTGA TTCCA TTCTG TTCTA TTCCA TTCAA TTCTT TTTCA TTCCA TTCGA ATCCT 1601
TTCTA TTGCA GTCCA TTCCA TTCGA GTCCA TTCCA ATCCC TTCCA TTCCA TTCCA TTACA GTCCA TTCCA 1671
ATAGA TTCCA TTCCT TTGCC TTCCA TTCGA ATCCA TTCCA TTCTA GTCCA TTCCA TTTGA GTCAA TTCCA 1741
TTCCA TTCCA TTCTA TTCCT TTCCA ATCCA TTCGA TTCCA TTCGA TTCAA TTCCA TTTGA TTCTC TTTCA 1811
TTCTA TTTTA TTCCA TGCCA TTTGA TTGCA TTGCA TTCCA TTCCG TTTGA TTCCA GTCCA TTCAA GAAAG 1881
TTCCA TTCCA GTCCA TTGCT TTCCA GTCCA TTCCA TTCCA CTCTA GTCTA TTCCA CTCCA TTCCT TTCCA 1951
TTCCA TTCCA TACTA TTCCA TTCCA TTCCT TTGCA TTCCG T---- TTCCA ATCTA TTCGA GTCCA TTGCA 2017
TTCCA GTCCA ATCCA TTCGA TTACA TTCCT TTTGA TTCCC TGCCA GTCGA TTGCA TTGCA TACTA GACCA 2087
TTCCA AAGGA GTCCA TTCCA TTCTA TCTCA ACACT TTCCA TTCCA CTCTG TTCGA GTCCA TTCCA TTCCA 2157
GTCCA TTTAA TTCAA GGGCA TTCCA TTCCA TTCCA TTCCA TTCCA TTTCA TATTA TTCCA TTCCA TTCAA 2227
TTCCA TTCCA GATGA TTCCA TTCCA TTCTA TACCA TTGCT CTCTG TTCCA TTCCA TTCCA TCTGT CTCCA 2297
TTCCT TTCGT TTCGA TTCCT TTCCA TTCCA TTCCA TTACA TTTGA TCCTA TTTTA TTAAA TTGCA TTCTA 2367
TTCGA GTGAT TTCCA TTCGA GTCCT TTCCA TTCGA TTCCA TTCCA TTCTA TTCCA TTCCT TTGGA TTCCA 2437
TTCCA TTCCG TTCCG TTCAC ATCAA TTCCT TGCGA TTCCA TTACA TTCGA TTTCT TGCCA TTCGA TTCCA 2507
TTCCT TTTGA CTCCA TTTCA TTCGA TTCCA TTCCA TTCCA TTAAT TTCCA AGTCC ----A TTCTA TTCCA 2573
GTCCA TTAGT TTCGA CTCCA TTGCA TTCGA GTGCA TTCCA TTCCG TGGCT GTCCA TTCCA TTCCG TTTGA 2643
TGCCA TTCCA TACGA TTCCA TTCAA TTCGA GACCA TTCTA TTCCT GTCCA TTCCT TGTGG TTCGA TTCCA 2713
TTTCA CTCTA GTCCA TTCCA TTCCA TTCAA TTCCA TTCGA CTCTA TTCCG TTCCA TTCAA TTCCA TTCCA 2783
TTCGA TTCCA TTTTT TTCGA GAACC TTCCA TTACA CTCCC TTCCA TTCCA GTGCA TTCCA TTCCA GTCTC 2853
TTCAG TTCGA TTCCA TTCCA TTCGT TTCGA TTCCT TTCCA TTCCA GCCCA TTCCA TTCCA TTCCA TTCCT 2923
TTCCT TTCCG TTTCA TTAGA TTCCA TTGCA TTCGA TTCCA TTCAA TTCAA TTCCG TGCTA TTCAA TTTGA 2993
TTCAT TTCCA TTTAA TTCCA TTCCA TTAGA TTCCA TTCCG TACGA TTCCA TTCCT TTTGA ATCCA TTCCA 3063
TTGGA GTCCA TTCAC TTCCA GAACA TTCCA TTCCA GTCGA ATCCA TTCGA GTACA TTCCA TTAAA GTTCA 3133
TTACA TTCTA ATACA TTCCA TTCCA TTGCA TTCCA TTCCA TTCCA TTCGA TGCCA TTCGA TTCCA TTCCA 3203
TGCCA AATCA TTGCA TTCCT TTCCA TTCCG TTCCT ATCAA TTCCA TTCCA TTCGA TTTAG TTCGA TTCTA 3273
TTCAC TTCCA TTCCA TTCGA TTCCA TTCCA TTGGA GTCAA TTCCT TTCGA CACCC AGCCT TTCCA GTCAA 3343
TGATT TTGGA TTCCA TTTTT TTGCA TTCCA TTACA TTCTA TGACA TTCGA TTCCG TTTCA TTGCA TTCCA 3413
TTCCA TACAT TTTTA TTCCA TTCGA GACCG TAGCA TTCCA CTTTA TTCCA GG---                   3465
```
