# Supplementary material for: Fate of the human Y chromosome linked genes and loci in prostate cancer cell lines DU145 and LNCaP
Source: BMC Genomics. 2013 May 11;14:323. doi: 10.1186/1471-2164-14-323 (PMC3660188; doi:10.1186/1471-2164-14-323)
Supplement: Additional file 3 — Multiple Sequence Alignment of 3.56 Kb sequence of DYZ1 array from DU145 and LNCaP with sequence from the NCBI database (Accession no. AC068123.5, Gene ID- 100499443). Perfectly aligned sequences are indicated by star and deletions, by hyphens. Mismatched base pairs, deletions or insertions are highlighted in green colour. [file 1471-2164-14-323-S3.pdf]

Additional file 4: Multiple Sequence Alignment of 3.4 Kb sequence of *DYZ1* array from DU145 and LNCaP with sequence from the NCBI database (Accession no. AC068123.5, Gene ID- 100499443). Perfectly aligned sequences are indicated by star and deletions, by hyphens. Mismatched base pairs, deletions or insertions are highlighted in green color.

```
DYZ1_DU145          CCTGTCCATTACACTACATTCCCTTCCATTCCAATGAATTCCATTCCATTCCAATCCATT 60
DYZ1_LNCaP          CCTGTCCATTACACTACATTCCCTTCCATTCCAATGAATTCCATTCCATTCCAATCCATT 60
DYZ1_AC068123.5     CCTGTCCATTACACTACATTCCCTTCCATTCCAATGAATTCCATTCCATTCCAATCCATT 60
                    ************************************************************

DYZ1_DU145          CCTTTCCTTTCGCTTGCATTCCATTCTATTCCCTTCTACTGCATACAATTTCACTCCATT 120
DYZ1_LNCaP          CCTTTCCTTTCGCTTGCATTCCATTCTATTCCCTTCTACTGCATACAATTTCACTCCATT 120
DYZ1_AC068123.5     CCTTTCCTTTCGCTTGCATTCCATTCTATTCTCTTCTACTGCATACAATTTCACTCCATT 120
                    ******************************* ****************************

DYZ1_DU145          CGTTCCCATTCCATTCAATTCCATTCCATTCAATTCCATTCCATTTGTTTCCATTCTCTT 180
DYZ1_LNCaP          CGTTCCCATTCCATTCAATTCCATTCCATTCAATTCCATTCCATTTGTTTCCATTCTCTT 180
DYZ1_AC068123.5     CGTTCCCATTCCATTCAATTCCATTCCATTCAATTCCATTCCATTTGTTTCCATTCTCTT 180
                    ************************************************************

DYZ1_DU145          CGATTCCATTTCTTTATATTCCATGCCATTCGATTCCATTCTATTGGGTTGCATTACATT 240
DYZ1_LNCaP          CGATTCCATTTCTTTATATTCCATGCCATTCGATTCCATTCTATTGGATTGCATTACATT 240
DYZ1_AC068123.5     CGATTCCATTTCTTTATATTCCATGCCATTCGATTCCATTCTATTGGGTTGCATTACATT 240
                    *********************************************** ************

DYZ1_DU145          CGTGTTCATTCCATTCCAGACCATTCCATTTGACTCCATTCCTTTCGAGCCCTTTCAATT 300
DYZ1_LNCaP          CGTGTTCATTCCATTCCAGACCATTCCATTTGACTCCATTCCTTTCGAGCCCTTTCAATT 300
DYZ1_AC068123.5     CGTGTTCATTCCATTCCAGACCATTCCATTTGACTCCATTCCTTTCGAGCCCTTTCAATT 300
                    ************************************************************

DYZ1_DU145          TGAGTCCATTCCTTTCCAGTCCATTTCACTCCAGTCCATTACTATCCATTCCATACCATT 360
DYZ1_LNCaP          TGAGTCCATTCCTTTCCAGTCCATTTCACTCCAGTCCATTACTATCCATTCCATACCATT 360
DYZ1_AC068123.5     TGAGTCCATTCCTTTCCAGTCCATTTCACTCCAGTCCATTACTATCCATTCCATACCATT 360
                    ************************************************************

DYZ1_DU145          CCATCCCATTCCATTCCATTCCATTCCATTCCATTCCATTCCATTCCATTCCATTCCATT 420
DYZ1_LNCaP          CCATCCCATTCCATTCCATTCCATTCCATTCCATTCCATTCCATTCCATTGCATTCAATT 420
DYZ1_AC068123.5     CCATCCCATTCCATTCCATTCCATTCCATTCCATTCCATTCCATTCCATTGCATTCCATT 420
                    ************************************************** ***** ***

DYZ1_DU145          CCATTCCATTCCATTGCACTGCACTCCATTCCATTACATTCTACTCTATCTGAGTCGATT 480
DYZ1_LNCaP          CCATTCCCTTCCATTGCACTGCACTCCATTCCATTACATTCTACTCTATCTGAGTCGATT 480
DYZ1_AC068123.5     CCATTCCATTCCATTGCACTGCACTCCATTCCATTACATTCTACTCTATCTGAGTCGATT 480
                    ******* ****************************************************

DYZ1_DU145          TTATTGCATTAGATTCTATTCCATTGGATTACTTTCCATTCGATTACATTCCATTCATGT 540
DYZ1_LNCaP          TTATTGCATTAGATTCTATTCCATTGGATTACTTTCCATTCGATTACATTCCATTCATGT 540
DYZ1_AC068123.5     TTATTGCATTAGATTCTATTCCATTGGATTACTTTCCATTCGATTACATTCCATTCATGT 540
                    ************************************************************

DYZ1_DU145          ACATTCCATTCCAGTCAATTACATTCGAGTTCATTACGTTACATTCCAGTATATTCCATT 600
DYZ1_LNCaP          ACATTCCATTCCAGTCAATTACATTCGAGTTCATTACATTACATTCCAGTATATTCCATT 600
DYZ1_AC068123.5     ACATTCCATTCCAGTCAATTACATTCGAGTTCATTACGTTACATTCCTGTATATTCCATT 600
                    ************************************* ********* ************

DYZ1_DU145          GTATTCGATCCCATTCCTTTCAATTCCATTTCATTCGACTCCATTATATTCAATTCCATT 660
DYZ1_LNCaP          GTATTCGATCCCATTCCTTTCAATTCCATTTCATTCGACTCCATTATATTCGATTCCATT 660
DYZ1_AC068123.5     GTATTCGATCCCATTCCTTTCAATTCCATTTCATTCGACTCCATTATATTCAATTCCATT 660
                    *************************************************** ********
```

```
DYZ1_DU145           CCACTCGAATCCATTCCATTAGAGGACATTCCATTCCAATGCATTCCATTCCATTCCATA 720
DYZ1_LNCaP           CCACTCGAATCCATTCCATTAGAGGACATTCCATTCCAATGCATTCCATTCCATTCCATA 720
DYZ1_AC068123.5      CCACTCGAATCCATTCCATTAGAGGACATTCCATTCCAATGCATTCCATTCCATTCCATA 720
                     ************************************************************

DYZ1_DU145           GCATTCCATTGCATTCGATTCCATTCCATTTGATGCCATTCCATTTGATGCCATTCCATG 780
DYZ1_LNCaP           GCATTCCATTGCATTCGATTCCATTCCATTTGATGCCATTCCATTTGATGCCATTCCATG 780
DYZ1_AC068123.5      GCATTCCATTGCATTCGATTCCATTCCATTTGATGTCATTCCATTTGATGCCATCCCATG 780
                     *********************************** ****************** *****

DYZ1_DU145           ACATTCCATTC-ATTCGAGTCCATTCCGTTCCAATTCATTCCATTCCGTTTCATGAAATT 839
DYZ1_LNCaP           ACATTCCATTCCATTCGAGTCCATTCCGTTCCAATTCATTCCATTCCGTTTCATGAAATT 840
DYZ1_AC068123.5      ACATTCCATTCCATTCGAGTCCATTCCGTTCCAATTCATTCCATTCCGTTTCATGAAATT 840
                     *********** ************************************************

DYZ1_DU145           CGAGTCCTTTCCAGTACATTTCATTCCAATCCCATCCAATCCAATCTACTCCATTCAATT 899
DYZ1_LNCaP           CGAGTCCTTTCCAGTACATTTCATTCCAATCCCATCCAATCCCATCTACTCCATTCAATT 900
DYZ1_AC068123.5      CGAGTCCTTTCCAGTACATTTCATTCCAATCCCATCCAATCCAATCTACTCCATTCAATT 900
                     ****************************************** *****************

DYZ1_DU145           CCTTTCCATTCCATTTGATTTGATTCCATTGATTTGATTCCATTCAGTTTGATTCCATTC 959
DYZ1_LNCaP           CCTTTCCATTCCATTTGATTTGATTCCATTGATTTGATTCCATTCAGTTTGATTCCATTC 960
DYZ1_AC068123.5      CCTTTCCATTCCATTTGATTTGATTCCATTGATTTGATTCCATTCAGTTTGATTCCATTC 960
                     ************************************************************

DYZ1_DU145           CGTGAAATTTCGTTCCATTCTATTCCATTGCATTACTTTCCATTCAATTCCATTCCATTT 1019
DYZ1_LNCaP           CGTGAAATTTCGTTCCATTCTATTCCATTGCATTACTTTCCATTCAATTCCATTCCATTT 1020
DYZ1_AC068123.5      CGTGAAATTTCGTTCCATTCTATTCCATTGCATTACTTTCCATTCAATTCCATTCCATTT 1020
                     ************************************************************

DYZ1_DU145           CATTTCAGTCCATTCGCTTCCTTTCCTTTCGATTCAATTCCATTTGATTCCACTCCATTC 1079
DYZ1_LNCaP           CATTTCAGTCCATTCGCTTCCTTTCCTTTCGATTCAATTCCATTTGATTCCACTCCATTC 1080
DYZ1_AC068123.5      CATTTCAGTCCATTCGCTTCCTTTCCTTTCGATTCAATTCCATTTGATTCCACTCCATTC 1080
                     ************************************************************

DYZ1_DU145           TATGCGATTTCATTCCAATCGATTCAATTCCATTCGATGACATTCCTTTCGTTTCCATTC 1139
DYZ1_LNCaP           TATGCGATTTCATTCCAATCGATTCAATTCCATTCGATGACATTCCTTTCGTTTCCATTC 1140
DYZ1_AC068123.5      TATGCGATTTCATTCCAATCGATTCAATTCCATTCGATGACATTCCTTTCGTTTCCATTC 1140
                     ************************************************************

DYZ1_DU145           CATTCGAGTCCATTTAATTTGAGCATTCGTGTCCATTCTATTCGAGTCCATTCCATTACC 1199
DYZ1_LNCaP           CATTCGAGTCCATTTAATTTGAGCATTCGTGTCCATTCTATTCGAGTCCATTCCATTACC 1200
DYZ1_AC068123.5      CATTCGAGTCCATTTAATTTGAGCATTCGTGTCCATTCTATTCGAGTCCATTCCATTACA 1200
                     *********************************************************** 

DYZ1_DU145           GTCTATTCTATTCCCTTCCATTCCTGTTGATTCAATTTCATTCCCTTCCATTCGATTCCT 1259
DYZ1_LNCaP           GTCTATTCTATTCCCTTCCATTCCTGTTGATTCAATTTCATTCCCTTCCATTCGATTCCT 1260
DYZ1_AC068123.5      GTCTATTCTATTCCCTTCCATTCGTGTTGATTCAATTTCATTCCCTTCCATTCGATTCCT 1260
                     *********************** ************************************

DYZ1_DU145           TTCCATTCGATTCCATTCCTTTCCATTCCATTCCATTCGTTCCCATTCCATGTGATTTCA 1319
DYZ1_LNCaP           TTCCATTCGATTCCATTCCTTTCCATTCCATTCCATTCGTTCCCATTCCATGTGATTTCA 1320
DYZ1_AC068123.5      TTCCATTCGATTCCATTCCTTTCCATTCCATTCCATTCGTTCCCATTCCATGTGATTTCA 1320
                     ************************************************************

DYZ1_DU145           TTCCATTCCAGTCCATTATATTCGAGTCCACTCCACTCCATTCTATTACATTCAATTCCT 1379
DYZ1_LNCaP           TTCCATTCCAGTCCATTATATTCGAGTCCACTCCACTCCATTCTATTACATTCAATTCCT 1380
DYZ1_AC068123.5      TTCCATTCCAGTCCATTATATTCGAGTCCACTCCACTCCATTCTATTACATTCAATTCCT 1380
                     ************************************************************

DYZ1_DU145           TTTGAGTCCGTTCCATAACACTCCATTCATTTCGATTCCATTTCTTGCCAGTTTTCTTCC 1439
DYZ1_LNCaP           TTTGAGTCCGTTCCATAACACTCCATTCATTTCGATTCCATTTCTTGCCAGTTTTCTTCC 1440
DYZ1_AC068123.5      TTTGAGTCCGTTCCATAACACTCCATTCATTTCGATTCCATTTCTTGCCAGTTTTCTTCC 1440
                     ************************************************************
```

```
DYZ1_DU145            ATTTTATTCCATTCCGTTCGATTCCATTCCATTCGATTGCATTCCATTCGAATCCTTTCC 1499
DYZ1_LNCaP            ATTTTATTCCATTCCGTTCGATTCCATTCCATTCGATTGCATTCCATTCGAATCCTTTCC 1500
DYZ1_AC068123.5       ATTTTATTCCATTCCGTTCGATTCCATTCCATTCGATTGCATTCCATTCGAATCCTTTCC 1500
                      ************************************************************

DYZ1_DU145            ATTCCATTTCATTCCATTCCTTTCTATTCCATTCCATTTCATTCGATTTGATTCCATTCT 1559
DYZ1_LNCaP            ATTCCATTTCATTCCATTCCTTTCTATTCCATTCCATTTCATTCGATTTGATTCCATTCT 1560
DYZ1_AC068123.5       ATTCCATTTCATTCCATTCCTTTCTATTCCATTCCATTTCATTCGATTTGATTCCATTCT 1560
                      ************************************************************

DYZ1_DU145            GTTCTATTCCATTCAATTCTTTTTCATTCCATTCGAATCCTTTCTATTGCAGTCCATTCC 1619
DYZ1_LNCaP            GTTCTATTCCATTCAATTCTTTTTCATTCCATTCGAATCCTTTCTATTGCAGTCCATTCC 1620
DYZ1_AC068123.5       GTTCTATTCCATTCAATTCTTTTTCATTCCATTCGAATCCTTTCTATTGCAGTCCATTCC 1620
                      ************************************************************

DYZ1_DU145            ATTCGAGTCCATTCCAATCCCTTCCATTCCATTCCATTAGCAGTCCATTCCAATAGATTC 1679
DYZ1_LNCaP            ATTCGAGTCCATTCCAATCCCTTCCATTCCATTCCATTA-CAGTCCATTCCAATAGATTC 1679
DYZ1_AC068123.5       ATTCGAGTCCATTCCAATCCCTTCCATTCCATTC----------------CAATAGATTC 1664
                      **********************************                **********

DYZ1_DU145            CATTCCTTTGCCTTCCATTCGAATCCATTCCATTCTAGTCCATTCCATTTGAGTCAATTC 1739
DYZ1_LNCaP            CATTCCTTTGCCTTCCATTCGAATCCATTCCATTCTAGTCCATTCCATTTGAGTCAATTC 1739
DYZ1_AC068123.5       CATTCCTTTGCCTTCCATTCGAATCCATTCCATTCTAGTCCATTCCATTTGAGTCAATTC 1724
                      ************************************************************

DYZ1_DU145            CATTCCATTCCATTCTATTCCTTTCCAATCCATTCGATTCCATTCGATTCAATTCCATTT 1799
DYZ1_LNCaP            CATTCCATTCCATTCTATTCCTTTCCAATCCATTCGATTCCATTCGATTCAATTCCATTT 1799
DYZ1_AC068123.5       CATTCCATTCCATTCTATTCCTTTCCAATCCATTCGATTCCATTCGATTCAATTCCATTT 1784
                      ************************************************************

DYZ1_DU145            GATTCTCTTTCATTCTATTTTATTCCATGCCATTTGATTGCATTGCATTCCATTCCGTTT 1859
DYZ1_LNCaP            GATTCTCTTTCATTCTATTTTATTCCATGCCATTTGATTGCATTGCATTCCATTCCGTTT 1859
DYZ1_AC068123.5       GATTCTCTTTCATTCTATTTTATTCCATGCCATTTGATTGCATTGCATTCCATTCCGTTT 1844
                      ************************************************************

DYZ1_DU145            GATTCCAGTCCATTCAAGAAAGTTCCATTCCAGTCCATTGCTTTCGAGTCCATTCCATTC 1919
DYZ1_LNCaP            GATTCCAGTCCATTCAAGAAAGTTCCATTCCAGTCCATTGCTTTCCAGTCCATTCCATTC 1919
DYZ1_AC068123.5       GATTCCAATCCATCCAAGAAAGTTCCATTCCAGTCCATTGCTTTCCAGTCCATTCCATTC 1904
                      ******* ***** ******************************* **************

DYZ1_DU145            CACTCTAGTCTATTCCACTCCATTCCTTTCCATTCCATTCCATACTATTCCATTCCATTC 1979
DYZ1_LNCaP            CACTCTAGTCTATTCCACTCCATTCCTTTCCATTCCATTCCATACTATTCCATTCCATTC 1979
DYZ1_AC068123.5       CACTCTAGTCTATTCCACTCCATTCCCTTCCATTGCATTCCATACTATTCCATTCCATTC 1964
                      ************************** ******* *************************

DYZ1_DU145            CTTTGCATTCCGTTTCCAATCTATTCGAGTCCATTGCATTCCAGTCCAGTCCATTCCATT 2039
DYZ1_LNCaP            CTTTGCATTCCGTTTCCAATCTATTCGAGTCCATTGCATTCCAGTCCAATCCATTCGATT 2039
DYZ1_AC068123.5       CTTTGCATTCCGTTTCCAATCTCTTCGAGTCCATTGCATTCCAGTCCAATCCATTCGATT 2024
                      ********************** ************************* ******* ***

DYZ1_DU145            ACATTCCTTTTGATTCCCTGCCAGTCGATTGCATTGCATACTACACCATTCCAAAGGAGT 2099
DYZ1_LNCaP            ACATTCCTTTTGATTCCCTGCCAGTCGATTGCATTGCATACTAGACCATTCCAAAGGAGT 2099
DYZ1_AC068123.5       ACATTCCTTTTGATTCCCTGCCAGTCGATTGCATTGCATACTACACCATTCCAAAGGAGT 2084
                      ******************************************* ****************

DYZ1_DU145            CCATTCCATTCTATCTCAACACTTTCCATTCCACTCTGTTCGAGTCCATTCCATTCCAGT 2159
DYZ1_LNCaP            CCATTCCATTCTATCTCAACACTTTCCATTCCACTCTGTTCGAGTCCATTCCATTCCAGT 2159
DYZ1_AC068123.5       TCATTCCATTCTATCTCCACACTTTCCATTCCACTCTGTTTGAGTCCATTCCATTCCAGT 2144
                       **************** ********************** *******************

DYZ1_DU145            CCATTTAATTCAAGGGCATTCCATTCCATTCCATTCCATTCCATTTCATATTATTCCATT 2219
DYZ1_LNCaP            CCATTTAATTCAAGGGCATTCCATTCCATTCCATTCCATTCCATTTCATATTATTCCATT 2219
DYZ1_AC068123.5       CCATTTAATTCAAGGGCATTCCATTCCATTCCATTCCATTCCATTTCATATTATTCCATT 2204
                      ************************************************************
```

```
DYZ1_DU145          CCATTCAATTCCATTCCAGATGATTCCATTCCATTCTATACCATTGCTCTCTGTTCCATT 2279
DYZ1_LNCaP          CCATTCAATTCCATTCCAGATGATTCCATTCCATTCTATACCATTGCTCTCTGTTCCATT 2279
DYZ1_AC068123.5     CCATTCAATTCCATTCCAGATGATTCCATTCCATTCTATACCATTGCTCTCTGTTCCATT 2264
                    ************************************************************

DYZ1_DU145          CCATTCCATCTGTCTCCATTCCTTTCGTTTCGATTCCTTTCCATTCCATTCCATTACATT 2339
DYZ1_LNCaP          CCATTCCATCTGTCTCCATTCCTTTCGTTTCGATTCCTTTCCATTCCATTCCATTACATT 2339
DYZ1_AC068123.5     CCATTCCATCTGTCTCCATTCCTTTCGTTTCGATTCCTTTCCATTCCATTCCATTACATT 2324
                    ************************************************************

DYZ1_DU145          TGATCCTATTTTATTAAATTGCATTCTATTCGAGTGATTTCCATTCGAGTCCTTTCCATT 2399
DYZ1_LNCaP          TGATCCTATTTTATTAAATTGCATTCTATTCGAGTGATTTCCATTCGAGTCCTTTCCATT 2399
DYZ1_AC068123.5     TGATCCTATTTTATCAAATTGCATTCTATTCGAGTGATTTCCATTCGAGTCCTTTCCATT 2384
                    ************** *********************************************

DYZ1_DU145          CGATTCCATTCCATTCTATTCCATTCCTTTGGATTCCATTCCATTCCGTTCCGTTCACAT 2459
DYZ1_LNCaP          CGATTCCATTCCATTCTATTCCATTCCTTTGGATTCCATTCCATTCCGTTCCGTTCACAT 2459
DYZ1_AC068123.5     CGATTCCATTCCATTCTATTCCATTCCTTTGGATCCCATTCCATTCCGTTCCGTTCACAT 2444
                    ********************************** *************************

DYZ1_DU145          CAATTCCTTGCGATTCCATTACATTCGATTTCTTGCCATTCGATTCCATTCCTTTTGACT 2519
DYZ1_LNCaP          CAATTCCTTGCGATTCCATTACATTCGATTTCTTGCCATTCGATTCCATTCCTTTTGACT 2519
DYZ1_AC068123.5     CAATTCCTGGCGATTCCATTACATTCGATTTCTTGCCATTCGATTCCATTCCTTTTGACT 2504
                    ******** ***************************************************

DYZ1_DU145          CCATTTCATTCGATTCCATTCCATTCCATTAATTTCCATTCCATTCGAGACCTTTCCATT 2579
DYZ1_LNCaP          CCATTTCATTCGATTCCATTCCATTCCATTAATTTCCA---------------------- 2557
DYZ1_AC068123.5     CCATTTCATTCGATTCCATTCCATTCCATTAATTTCCATTCCATTCGAGACCTTTCCATT 2564
                    **************************************

DYZ1_DU145          GCAGTCTTTTCCCTTCGAGTCCATTCCGTTCGATTCCCTTCC---------------ATT 2624
DYZ1_LNCaP          ------------------------------------------------------------
DYZ1_AC068123.5     GCAGTCTTTTCCCTTCGAGTCCATTCCGTTCGATTCCCTTCCGTTCGATTCCCTTCCATT 2624

DYZ1_DU145          CGATTCCATTCCATTGGAGTCCGTACCAGTCGAGTCCATTCTATTCCAGTCCATTAGTTT 2684
DYZ1_LNCaP          --------------------------------AGTCCATTCTATTCCAGTCCATTAGTTT 2585
DYZ1_AC068123.5     CGATTCCATTCCATTGGAGTCCGTACCAGTCGAGTCCATTCTATTCCAGTCCATTAGTTT 2684
                                                    ****************************

DYZ1_DU145          CGACTCCATTGCATTCGAGTGCATTCCATTCCGTGGCTGTCCATTCCATTCCGTTTGATG 2744
DYZ1_LNCaP          CGACTCCATTGCATTCGAGTGCATTCCATTCCGTGGCTGTCCATTCCATTCCGTTTGATG 2645
DYZ1_AC068123.5     CGACTCCATTGCATTCGAGTGCATTCCATTCCGTGGCTGTCCATTCCATTCCGTTTGATG 2744
                    ************************************************************

DYZ1_DU145          CCATTCCATACGATTCCATTCAATTCGAGACCATTCTATTCCTGTCCATTCCTTGTGGTT 2804
DYZ1_LNCaP          CCATTCCATACGATTCCATTCAATTCGAGACCATTCTATTCCTGTCCATTCCTTGTGGTT 2705
DYZ1_AC068123.5     CCATTCCATACGATTCCATTCAATTCGAGACCATTCTATTCCTGTCCATTCCTTGTGGTT 2804
                    ************************************************************

DYZ1_DU145          CGATTCCATTTCACTCTAGTCCATTCCATTCCATTCAATTCCATTCGACTCTATTCCGTT 2864
DYZ1_LNCaP          CGATTCCATTTCACTCTAGTCCATTCCATTCCATTCAATTCCATTCGACTCTATTCCGTT 2765
DYZ1_AC068123.5     CGATTCCATTTCACTCTAGTCCATTCCATTCCATTCAATTCCATTCGACTCTATTCCGTT 2864
                    ************************************************************

DYZ1_DU145          CCATTCAATTCCATTCCATTCGATTCCATTTTTTTCGAGAACCTTCCATTACACTCCCTT 2924
DYZ1_LNCaP          CCATTCAATTCCATTCCATTCGATTCCATTTTTTTCGAGAACCTTCCATTACACTCCCTT 2825
DYZ1_AC068123.5     CCATTCAATTCCATTCCATTCGATTCCATTATTTTCGAGAACCTTGCATTACACTCCCTT 2924
                    ****************************** ************** **************

DYZ1_DU145          CCATTCCAGTGCATTCCATTCCAGTCTCTTCAGTTCGATTCCATTCCATTCGTTTCGATT 2984
DYZ1_LNCaP          CCATTCCAGTGCATTCCATTCCAGTCTCTTCAGTTCGATTCCATTCCATTCGTTTCGATT 2885
DYZ1_AC068123.5     CCATTCCAGTGCATTCCATTCCAGTCTCTTCACTTCGATTCCATTCCATTCGTTTCGATT 2984
                    ******************************** ***************************
```

```
DYZ1_DU145          CCTTTCCATTCCAGCCCATTCCATTCCATTCCATTCCTTTCCTTTCCGTTTCATTAGATT 3044
DYZ1_LNCaP          CCTTTCCATTCCAGCCCATTCCATTCCATTCCATTCCTTTCCTTTCCGTTTCATTAGATT 2945
DYZ1_AC068123.5     CCTTTCCATTCCAGCCCATTCCATTCCATTCCATTCCTTTCCTTTCCGTTTCATTAGATT 3044
                    ************************************************************

DYZ1_DU145          CCATTGCATTCGATTCCATTCAATTCAATTCCGTGCTATTCAATTTGATTCATTTCCATT 3104
DYZ1_LNCaP          CCATTGCATTCGATTCCATTCAATTCAATTCCGTGCTATTCAATTTGATTCATTTCCATT 3005
DYZ1_AC068123.5     CCATTGCATTCGATTCCATTCAATTCAATTCCGTGCTATTCAATTTGATTCATTTCCATT 3104
                    ************************************************************

DYZ1_DU145          TAATTCCATTCCATTAGATTCCATTCCGTACGATTCCATTCCTTTTGAATCCATTCCATT 3164
DYZ1_LNCaP          TAATTCCATTCCATTAGATTCCATTCCGTACGATTCCATTCCTTTTGAATCCATTCCATT 3065
DYZ1_AC068123.5     TAATTCCATTCCATTAGATTCCATTCCGTACGATTCCATTCCTTTTGAATCCATTCCATT 3164
                    ************************************************************

DYZ1_DU145          GGAGTCCATTCACTTCCAGAACATTCCATTCCAGTCGAATCCATTCGAGTACATTCCATT 3224
DYZ1_LNCaP          GGAGTCCATTCACTTCCAGAACATTCCATTCCAGTCGAATCCATTCGAGTACATTCCATT 3125
DYZ1_AC068123.5     GGAGTCCATTCACTTCCAGAACATTCCATTCCAGTCGAATCCATTCGAGTACATTCCATT 3224
                    ************************************************************

DYZ1_DU145          AAAGTTCATTACATTCTAATACATTCCATTCCATTGCATTCCATTCCATTCCATTCGATG 3284
DYZ1_LNCaP          AAAGTTCATTACATTCTAATACATTCCATTCCATTGCATTCCATTCCATTCCATTCGATG 3185
DYZ1_AC068123.5     AAAGTTCATTACATTCTAATACATTCCATTCCATTGCATTCCATTCCATTCCATTCGATG 3284
                    ************************************************************

DYZ1_DU145          CCATTCGATTCCATTCCATGCCAAATCATTGCATTCCTTTCCATTCCGTTCCTATCAATT 3344
DYZ1_LNCaP          CCATTCGATTCCATTCCATGCCAAATCATTGCATTCCTTTCCATTCCGTTCCTATCAATT 3245
DYZ1_AC068123.5     CCATTCGATTCCATTCCATGCCAAATCATTGCATTCCTTTCCATTCCGTTCCTATCAATT 3344
                    ************************************************************

DYZ1_DU145          CCATTCCATTCGATTTAGTTCGATTCTATTCACTTCCATTCCATTCGATTCCATTCCATT 3404
DYZ1_LNCaP          CCATTCCATTCGATTTAGTTCGATTCTATTCACTTCCATTCCATTCGATTCCATTCCATT 3305
DYZ1_AC068123.5     CCATTCCATTCGATTTAGTTCGATTCTATTCACTTCCATTCCATTCGATTCCATTCCATT 3404
                    ************************************************************

DYZ1_DU145          GGAGTCAATTCCTTTCGACACCCAGCCTTTCCAGTCAATGATTTTGGATTCCATTTTTTT 3464
DYZ1_LNCaP          GGAGTCAATTCCTTTCGACACCCAGCCTTTCCAGTCAATGATTTTGGATTCCATTTTTTT 3365
DYZ1_AC068123.5     GGAGTCAATTCCTTTCGACACCCAGCCTTTCCAGTCAATGATTTTGGATTCCATTTTTTT 3464
                    ************************************************************

DYZ1_DU145          GCATTCCATTACATTCTATGACATTCGATTCCGTTTCATTGCATTCCATTCCATACATTT 3524
DYZ1_LNCaP          GCATTCCATTACATTCTATGACATTCGATTCCGTTTCATTGCATTCCATTCCATACATTT 3425
DYZ1_AC068123.5     GCATTCCATTACATTCTATGACATTCGATTCCGTTTCATTGCATTCCATTCCATACATTT 3524
                    ************************************************************

DYZ1_DU145          TTATTCCATTCGAGACCGTAGCATTCCACTTTATTCCAGG 3564
DYZ1_LNCaP          TTATTCCATTCGAGACCGTAGCATTCCACTTTATTCCAGG 3465
DYZ1_AC068123.5     TTATTCCATTCGAGACCGTAGCATTCCACTTTATTCCAGG 3564
                    ****************************************
```
